# Virtual Mental Health Care and Suicide-Related Events

**DOI:** 10.1001/jamanetworkopen.2024.43054

**Published:** 2024-11-05

**Authors:** Kertu Tenso, Kiersten Strombotne, Melissa M. Garrido, Jessica Lum, Steven Pizer

**Affiliations:** 1 Department of Health Law Policy and Management, Boston University School of Public Health, Boston, Massachusetts; 2 Partnered Evidence-Based Policy Resource Center, VA Boston Healthcare System, Boston, Massachusetts

## Abstract

**Importance:**

The rising suicide rates in the US emphasize the need for effective prevention. While telehealth has transformed access to mental health care, the impact of telehealth on suicide outcomes is unknown.

**Objective:**

To evaluate the association of virtual mental health services with individual-level suicide-related events (SREs).

**Design, Setting, and Participants:**

This retrospective cohort study using broadband access as an instrumental variable assessed a national sample of Veterans Health Administration patients who received mental health care between March 1, 2020, and December 31, 2021. Participants were recently separated (ie, discharged or released from active duty) veterans who completed their active duty service between March 1, 2019, and December 31, 2020, and who received at least 2 outpatient or inpatient diagnoses related to major depressive disorder, substance use disorder, or posttraumatic stress disorder within the year before their most recent separation date. Data were analyzed May 1 to October 31, 2023.

**Exposure:**

Percentage of a patient’s total mental health visits that were conducted virtually by psychiatrists, psychologists, or social workers within a calendar month.

**Main Outcomes and Measures:**

Binary measure indicating whether the patient had experienced an SRE (defined as a nonfatal suicide attempt, intentional self-harm, or suicide death) in a specific month and year as evaluated an instrumental variable probit model.

**Results:**

The sample included 66 387 data points from 16 236 unique recently separated veterans. Among these entries, 44 766 were for male veterans (67.4%), the mean (SD) age across the sample was 32.9 (8.9) years, and the sample was representative of the US veteran population. There were 929 SREs (1.4%). Virtual mental health visits comprised a mean (SD) of 44.6% (46.1%) of all mental health visits. In instrumental variable probit analyses accounting for factors simultaneously associated with use of virtual mental health care and SRE risk, a 1% increase in the probability of virtual mental health visits was associated with a 2.5% decrease in SREs.

**Conclusions and Relevance:**

Findings from this cohort study using a retrospective quasi-experimental design found that an increase in virtual mental health visits relative to total visits was associated with a statistically significant decrease in SREs, suggesting that providing virtual mental health services may reduce suicide-related outcomes.

## Key Points

**Question:**

Is the shift from face-to-face to virtual mental health service delivery associated with the risk of suicide-related events?

**Findings:**

In this cohort study assessing 66 387 data points from 16 236 unique veterans, a 1% increase in the percentage of virtual mental health visits relative to the total visits was associated with a statistically significant 2.5% decrease in suicide-related events.

**Meaning:**

The results of this cohort study suggest that offering virtual mental health care in addition to in-person care may reduce suicide-related events.

## Teaser

This cohort study assesses whether offering virtual mental health care in addition to in-person care is associated with the risk of suicide-related events among Veterans Health Administration patients with major depressive disorder, substance use disorder, or posttraumatic stress disorder.

## Introduction

Suicide remains a significant public health concern in the US, as the suicide rate increased by 35% between 2000 and 2018.^1^ In 2020, an estimated 12.2 million American adults seriously contemplated suicide, 3.2 million formed a suicide plan, and 1.2 million attempted suicide.^2^ However, suicide is often preventable through proactive strategies, including universal interventions addressing economic and social disparities, educational campaigns for mental health awareness, and targeted health care initiatives to screen high-risk populations.^3^

The rise of telehealth—a system for delivering health care remotely through technology—has reshaped the landscape of mental health services and suicide prevention. Telehealth is particularly suitable for mental health care because psychiatric diagnoses and treatments rely on discussions rather than physical assessments.^4^ Moreover, telehealth can reduce stigma, break down geographical barriers, reduce caregiver burden, and enhance access to mental health resources.^5,6,7,8^ Enhancing access is vital, particularly in rural regions with limited health care professional availability.^5^ The COVID-19 pandemic further highlighted the appropriateness of telemedicine in maintaining access to mental health services while enforcing safety and social distancing protocols.^9,10^ However, challenges persist, such as technological accessibility and the seamless integration of telehealth into the established health care infrastructure.^11,12,13^

Several studies have highlighted the effectiveness of mental telehealth. Randomized clinical trials have demonstrated that telepsychiatry treatment through videoconferencing has an equivalent efficacy to in-person psychiatric treatment in terms of psychiatric test scores, treatment adherence, and patient satisfaction.^14,15^ However, it is important to note that much of this evidence comes from small clinical trials, and while these studies show promising results, they do not fully establish the comparative effectiveness of video vs in-person mental health care. Trials across diverse patient groups further emphasize that video-assisted psychotherapy is as effective as traditional methods for treating conditions such as posttraumatic stress disorder (PTSD),^16,17,18,19^ depression,^20^ obsessive-compulsive disorder,^21^ social anxiety disorder,^22^ schizophrenia,^23^ and panic disorder.^24^ Moreover, patients and health care professionals are generally satisfied with virtual care.^25,26^

Nevertheless, caution may be warranted when considering mental telehealth for individuals with suicidal ideation. While remote sessions offer opportunities for the early detection of suicidal tendencies and collaborative safety planning involving household members,^27^ assessing and managing emergent safety concerns remotely can be challenging. A study involving 52 mental health professionals identified several perceived challenges of telehealth, with only about one-fifth endorsing its use for high-risk patients, thus highlighting general reservations about its application in individuals with suicidal ideation or behaviors.^28^

Despite the extensive literature on mental telehealth, there is a gap in our understanding of its direct effect on suicide outcomes. Most studies offer insights based on specific digital interventions and self-reported outcomes.^29^ A national descriptive study by the US Veterans Health Administration (VHA) showed that the total number of psychiatric hospitalizations fell after patient enrollment in telehealth services between 2006 and 2010.^30^ However, a gap remains in experimental and quasi-experimental studies, which have been suggested to demonstrate causal relationships and can inform policy and clinical decisions. Considering the increased use of mental telehealth by individuals with suicidal ideation or behaviors, understanding causal relationships between care modality and suicide-related outcomes is paramount.^31^

This evaluation explores whether virtual mental health service use is associated with individual-level suicide-related events (SREs). Specifically, is the shift from face-to-face to virtual mental health service delivery associated with the risk of SREs? The VHA is an ideal setting for this study because it is the largest integrated health care system in the US, has been at the forefront of mental telehealth innovations, and hosts one of the largest mental telehealth networks in the world.^32^ During the past decade, the VHA has significantly expanded its virtual care offerings, leveraging platforms such as VA Video Connect, initiatives such as Tablet to Home, and establishing dedicated TeleMental Health Hubs.^33^ Their swift response to the COVID-19 pandemic led to a staggering 556% surge in daily virtual mental health sessions by April 2020 compared with March 2020.^33,34^

## Methods

This cohort study was conducted as a quality improvement activity for the VHA and was deemed to not be research; thus, there was no research oversight committee involvement and this project was exempt from the requirement of obtaining informed consent. This report follows the Strengthening the Reporting of Observational Studies in Epidemiology (STROBE) cohort study reporting guideline and the Transparent Reporting of Evaluations With Nonrandomized Designs (TREND) reporting guideline.

### Evaluation Design

In an ideal evaluation design, we would randomize patients to various virtual care use groups to achieve the most accurate causal analysis, but doing so would be neither practical nor ethical. Therefore, we rely on observational data. However, determining the causal effects of telehealth on SREs using observational data is not possible because of the absence of randomization, the potential for reverse causality, and omitted variable bias. Reverse causality may occur if telehealth use is associated with underlying mental health needs, leading veterans to adapt their virtual mental health care use in response to changes in their mental health needs. Omitted variable bias can arise when our models exclude variables associated with both virtual care utilization and suicidal behaviors. For example, this problem may arise in patients experiencing anxiety so severe that they cannot travel for in-person clinic visits.

To address these concerns, we used a quasi-experimental instrumental variable (IV) probit model designed to address problems related to reverse causality and potential confounders when dealing with a binary outcome variable. For an instrument for virtual care to be considered valid in our IV model, it must meet 2 essential criteria. First, it needs to demonstrate relevance, indicating a strong association between exposure and the instrument, as determined through the application of the Kleibergen-Paap weak instruments test and comparison with the Stock-Yogo critical values.^35^ Second, the instrument should affect the outcomes of interest solely through the exposure variable, without any direct effects on the outcome, in accordance with the exclusion restriction.^36^ This can be both argued theoretically and supported by the falsification test included in eAppendix 4 in Supplement 1.

Our chosen instrument was the percentage of the population in a county with access to 3 or more internet providers offering broadband speeds of 100 megabits per second (Mbps) or higher. This instrument was selected based on its strong correlation with virtual care utilization, determined through an evaluation of various broadband configurations. Broadband internet access is strongly linked to virtual care because it serves as a prerequisite for virtual medical visits. However, there is no expectation that broadband internet speed and the number of available internet providers will directly influence SREs.

### Data and Population

The cohort consisted of recently separated (ie, discharged or released from active duty) veterans with a background of behavioral health diagnoses according to the Department of Veterans Affairs (VA) Infrastructure for Clinical Intelligence (DaVINCI) data. DaVINCI provides VA investigators with access to data from the Military Health System Data Repository, allowing them to add Department of Defense (DoD) data to VA data and enhance the comprehensiveness of patients’ health care history. We included veterans who had completed their active duty service between March 1, 2019, and December 31, 2020, and received at least 2 outpatient diagnoses or a single inpatient diagnosis related to major depressive disorder, substance use disorder, or PTSD within the year before their most recent separation date. A recently separated cohort was chosen to establish that mental health diagnoses occurred before the measurement of our treatment variable. Using the recently separated cohort, we had access to DoD data on preexisting mental health conditions. We combined patients with depression, PTSD, and substance use disorder to address the overall impact of virtual mental health care across a range of serious mental health conditions, aligning with prior published work.^37^ We followed up these individuals starting March 1, 2020, for up to 13 months after separation, including the month of separation. The decision to initiate the observation period in March 2020 was driven by our focus on the period after the beginning of the COVID-19 pandemic, which saw a significant increase in telehealth services. We limited the follow-up period to no more than 13 months postseparation due to the heightened risk experienced by patients during the period closest to their separation.^38^

The independent variable of interest was the individual-level percentage of mental health visits provided via virtual care (mental telehealth via video conferencing) by psychiatrists, psychologists, or social workers within a specific month. To obtain these data, we used Managerial Cost Accounting stop codes from the VA Corporate Data Warehouse (CDW) to narrow our sample to outpatient mental health encounters. We excluded observations with no mental health utilization because our objective was to investigate the association of virtual mental health care utilization with SREs.

The outcome variable was a binary measure indicating whether the patient had experienced an SRE (defined as a nonfatal suicide attempt, intentional self-harm, or suicide death) during the same calendar month of the visits. The data were sourced from 2 different origins, following research indicating that data accuracy is maximized when both types are used.^39,40^ First, we searched for relevant nonfatal suicide attempt *International Statistical Classification of Diseases, Tenth Revision* codes in the CDW outpatient, inpatient, and billing tables (eAppendix 3 in Supplement 1).^41^ Second, we obtained surveillance data from the VA Office of Mental Health and Suicide Prevention, which included information from 3 distinct surveillance sources: the Suicide Prevention Application Network, Comprehensive Suicide Risk Evaluation, and Suicidal Behavior and Overdose Report. For this evaluation, we included all SREs from these surveillance data sources.

Individual-level covariates obtained from the CDW electronic health records included the following: age, sex, race, ethnicity, priority status, marital status, distance from closest VHA primary care facility, and rurality of the patient’s residential address location (defined by Rural Urban Commuting Area codes). Race and ethnicity were assessed because race and ethnicity are well-documented factors associated with veterans' suicide disparities.^42^ Race was obtained from electronic health records and grouped into the following categories: American Indian or Alaska Native, Asian, Black or African American, Native Hawaiian or Other Pacific Islander, White, more than 1 race, and unknown (including declined to answer). Ethnicity was also obtained from electronic health records that captured whether a patient identified as Hispanic or Latino, with options being Hispanic or Latino, not Hispanic or Latino, and unknown or declined.^43^ We also gathered information about alternative insurance coverage and income in the facility catchment area (by creating a weighted mean of values associated with the counties in which VA-enrolled veterans resided) from the VA Survey of Enrollees.^44^ To identify quasi-experimental variations in virtual care utilization, we obtained data on broadband availability, speed, and number of internet service providers at the county level from the Federal Communications Commission.^45^ To control for local COVID-19 burden, we included county-month level cumulative COVID-19 mortality counts from Johns Hopkins Coronavirus Resource Center.^46^

Observations with missing or incomplete data were excluded to ensure data integrity. The detailed sample creation, missingness, and the inclusion and exclusion processes are outlined in eAppendices 1 and 2 in Supplement 1.

### Statistical Analysis

All models incorporated the following individual-level covariates: race, ethnicity, age, sex, priority status, marital status, rurality of the patient’s residential address location (defined by Rural Urban Commuting Area codes), and distance from closest VHA primary care facility. Furthermore, we accounted for the following facility-month level covariates: alternative insurance coverage (any non-VA health care coverage, either public or private) and per capita income in the facility catchment area. We used facility fixed effects to account for time-invariant characteristics unique to each facility and applied month fixed effects to control for seasonal variations that could potentially influence the outcome of interest. To address repeated observations, we used SEs clustered at the individual level.

We estimated a first-stage IV probit in which we regressed the percentage of mental health visits that were provided via virtual care in a given month on broadband and the aforementioned individual and facility-level covariates. We included month fixed effects to control for seasonal variation and facility fixed effects to control for facility time invariant characteristics. Then, we estimated the second stage of IV probit in which we regressed having an SRE on the percentage of mental health visits that were provided via virtual care, residuals from the first stage-estimation, and all the same individual and facility-level covariates. As in the first stage, we included month and facility fixed effects. In addition to the IV probit, we also estimated a single-equation probit model with SRE as the outcome, virtual care as an independent variable of interest, and controlling for all the same individual- and facility-level covariates, as well as month and facility fixed effects.

Two sensitivity analyses were performed. The first analysis was conducted to address the concern of treatment (virtual care) and outcome (SRE) being recorded in the same period, potentially having the SRE as a reason for the virtual care visit. We introduced a lead time for the SRE outcome variable and replicated our primary model with the SRE outcome from the following month. The second analysis aimed to address the concern regarding minimal variability in mental health visit types during the early months of the pandemic. For this analysis, we replicated the primary model once more, after omitting data from the early pandemic months of March, April, and May 2020.

Similar to Feyman et al,^37^ we examined via Heckman modeling whether there was a potential for unobserved factors to influence both the receipt of any VHA mental health care and SREs. eAppendix 5 in Supplement 1 provides the rationale and details of this analysis. All analyses were conducted from May 1 to October 31, 2023, with Stat/MP, version 16.0 (StataCorp LLC). A 2-sided value of *P* < .05 was considered statistically significant.

## Results

In our sample of 66 387 observations representing 16 236 unique veterans (mean [SD] age, 32.9 [8.9] years; 21 621 [32.6%] female and 44 766 [67.4%] male; 683 [1.0%] American Indian or Alaska Native, 2271 [3.4%] Asian, 17 250 [26.0%] Black, 1232 [1.9%] Native Hawaiian or Other Pacific Islander, 33 538 [50.5%] White, 1488 [2.2%] more than 1 race, 9925 [15.0%] race unknown; 9602 [14.5%] Hispanic, 45 897 [69.1%] non-Hispanic, and 10 888 [16.4%] ethnicity unknown; and 32 785 [49.4%] married), 929 of individual-month observations (1.4%) included an SRE. There was a mean (SD) of 2.2 (2.8) visits per month, with a mix of virtual, phone, and in-person visits (Table 1). Virtual mental health visits represented a mean (SD) of 44.6% (46.1%) of all mental health visits in our sample. The mean (SD) percentage of the county population with access to 3 or more internet providers offering broadband speeds of 100 Mbps or higher was 19.6% (23.0%). The mean (SD) driving distance to the nearest VHA primary care facility was 12.7 (10.8) miles.

**Table 1.** Descriptive Statistics of the Evaluation Sample (N = 66 387)

Variable	Observations, frequency (%)
Percentage of virtual mental health visits, mean (SD)	44.6 (46.1)
Percentage of suicide-related events, mean (SD)	1.4 (11.9)
No. total mental health visits per month, mean (SD)	2.2 (2.8)
Percentage of counties with ≥3 internet providers with ≥100/Mbps speed, mean (SD)	19.6 (23.0)
Drive distance to closest VHA primary care facility, mean (SD), miles	12.7 (10.8)
Age, mean (SD), y	32.9 (8.9)
Sex	
Female	21 621 (32.6)
Male	44 766 (67.4)
Race	
American Indian or Alaska Native	683 (1.0)
Asian	2271 (3.4)
Black	17 250 (26.0)
Native Hawaiian or Other Pacific Islander	1232 (1.9)
White	33 538 (50.5)
More than 1 race	1488 (2.2)
Race unknown	9925 (15.0)
Ethnicity	
Hispanic	9602 (14.5)
Non-Hispanic	45 897 (69.1)
Ethnicity unknown	10 888 (16.4)
Marital status	
Married	32 785 (49.4)
Single or never married	19 634 (29.6)
Divorced, separated, or widowed	10 772 (16.2)
Marital status unknown	3196 (4.8)
Priority status	
1-3 (Service-connected disability)	61 370 (92.4)
4-6	3992 (6.0)
7 and 8 (High income)	883 (1.3)
Missing	142 (0.2)
Rural area	
Not rural or islands	53 774 (81.0)
Rural	12 613 (19.0)
Health insurance coverage as proportion of facility enrollees, mean (SD)	
Comprehensive	0.66 (0.05)
Some	0.20 (0.05)
None	0.14 (0.03)
Unknown	0.002 (0.003)
Household income as proportion of facility enrollees, mean (SD), $	
<20 000	0.19 (0.04)
20 000 to <50 000	0.41 (0.05)
50 000 to <75 000	0.18 (0.03)
≥75 000	0.19 (0.07)
Income unknown	0.04 (0.01)
County-level COVID-19 mortality per 10 000, mean (SD)	8.0 (7.9)
Months since separation, mean (SD)	7.2 (3.4)

Abbreviation: Mbps, megabits per second.

The aforementioned demographic characteristics indicated that our sample was representative of the overall veteran population in the US. Additionally, 883 data points (1.3%) were for veterans classified under priority statuses 7 or 8 (typically assigned to higher-income veterans), and 12 613 data points (19.0%) represented veterans living in rural areas. Additional descriptive statistics on virtual care use by sex, race, and ethnicity are reported in eAppendix 6 in Supplement 1.

In terms of facility-related factors, the individuals in the sample resided closest to a primary care facility, where 66.4% of enrollees had comprehensive health insurance and 41.0% reported an annual household income between $20 000 and $50 000. In our sample, there was a mean (SD) of 8.0 (7.9) COVID-19 fatalities for every 10 000 people in a county.

In our single-equation probit models, an increase in virtual mental health visits was associated with a lower probability of suicide attempts, with a coefficient of −0.0032 (robust standard error, 0.0003; *P* < .001) (Table 2). The elasticity of SREs with respect to the proportion of virtual care visits was −0.41, meaning that a 1% increase in the probability of virtual mental health visits was associated with a 0.4% decrease in SREs (0.01 percentage points). Marginal effects of the single-equation probit model are reported in eAppendix 7 in Supplement 1.

**Table 2.** Probit and IV Probit Primary Results With Suicide-Related Event as the Dependent Variable^a^

Variable	Probit		IV probit			
			First stage		Second stage	
	Coefficient (robust SE)	*P* value	Coefficient (robust SE)	*P* value	Coefficient (robust SE)	*P* value
Percentage of virtual mental health visits	−0.0032 (0.0003)	<.001	NA	NA	−0.0161 (0.0049)	.001
Percentage of county with ≥3 internet providers with ≥100/Mbps speed	−0.0017 (0.0010)	.09	0.1021 (0.0161)	<.001	NA	NA
Age	−0.0261 (0.0029)	<.001	0.4143 (0.0363)	<.001	−0.0156 (0.0071)	.03
Sex						
Female	−0.0370 (0.0415)	.37	6.7969 (0.6436)	<.001	0.0621 (0.0577	.28
Male	1 [Reference]		1 [Reference]		1 [Reference]	
Race						
American Indian or Alaska native	0.2266 (0.1231)	.07	−4.6590 (2.7935)	.10	0.1208 (0.1189)	.31
Asian	−0.0367 (0.1090)	.74	−0.4983 (1.7408)	.77	−0.0365 (0.0905)	.69
Black	−0.0503 (0.0538)	.35	−3.1735 (0.7368)	<.001	−0.0838 (0.0442)	.06
Native Hawaiian or Other Pacific Islander	−0.2960 (0.2380)	.21	−2.6856 (2.4362)	.27	−0.2766 (0.1982)	.16
White	1 [Reference]	NA	1 [Reference]	NA	1 [Reference]	NA
More than 1	−0.1599 (0.1320)	.22	0.0223 (1.8373)	.99	−0.1294 (0.1120)	.25
Unknown	−0.0032 (0.0632)	.96	−1.5267 (0.9425)	.10	−0.0233 (0.0531)	.66
Ethnicity						
Hispanic	−0.0135 (0.0570)	.81	0.4431 (0.9117)	.63	−0.0050 (0.0486)	.92
Non-Hispanic	1 [Reference]		1 [Reference]	NA	1 [Reference]	NA
Unknown	−0.0312 (0.0583)	.59	−1.6263 (0.8884)	.07	−0.0474 (0.0484)	.33
Marital status						
Married	1 [Reference]		1 [Reference]	NA	1 [Reference]	NA
Single or never married	0.0699 (0.0469)	.14	−2.0432 (0.7498)	.006	0.0291 (0.0426)	.50
Divorced, separated or widowed	0.0773 (0.0556)	.16	−3.1171 (0.8514)	<.001	0.0205 (0.0556)	.71
Marital status unknown	0.0610 (0.0876)	.48	2.0596 (1.4483)	.16	0.0775 (0.0717)	.28
Priority status						
1-3 (Service-connected disability, no co-pays)	1 [Reference]		1 [Reference]	NA	1 [Reference]	NA
4-6 (No service-connected disability, no co-pays)	0.2250 (0.0614)	<.001	−7.5466 (1.1762)	<.001	0.0804 (0.0938)	.39
7-8 (No service-connected disability, have co-pays)	0.3250 (0.1101)	.003	−2.9528 (2.5928)	.25	0.2237 (0.1173)	.06
Priority status unknown	0.3133 (0.2253)	.16	−27.6397 (4.5607)	<.001	−0.1203 (0.2764)	.66
Rurality						
Not rural or islands	1 [Reference]	NA	1 [Reference]	NA	1 [Reference]	NA
Rural	0.0465 (0.0530)	.38	0.9195 (0.9130)	.31	0.0502 (0.0442)	.26
Drive distance to closest VHA primary care facility	−0.0008 (0.0017)	.64	0.0605 (0.0334)	.07	0.0002 (0.0015)	.92
Health insurance coverage as proportion of facility enrollees						
No insurance	1 [Reference]	NA	1 [Reference]	NA	1 [Reference]	NA
Comprehensive coverage	−1.2021 (1.9885)	.54	−122.3128 (31.9993)	<.001	−2.6333 (1.7419)	.13
Some coverage	−2.4381 (2.3921)	.31	−111.9930 (38.0256)	.003	−3.4965 (2.0099)	.08
Insurance unknown	3.0193 (13.6324)	.82	467.4954 (202.6018)	.02	8.7861 (11.5417)	.45
Household income as proportion of facility enrollees, $						
≥75 000	1 [Reference]	NA	1 [Reference]	NA	1 [Reference]	NA
<20 000	0.0859 (2.5169)	.97	−184.4566 (36.2317)	<.001	−2.4301 (2.4168)	.31
20 000 to <50 000	−2.9325 (2.1235)	.17	−96.6221 (33.4529)	.004	−3.6895 (1.7695)	.04
≥50 000 to <75 000	−2.5585 (2.0268)	.21	−38.9185 (30.5104)	.20	−2.6039 (1.6867)	.12
Income unknown	−0.6917 (3.4674)	.84	−228.7967 (58.3929)	<.001	−3.6621 (3.1201)	.24
Months since separation	−0.0067 (0.0050)	.18	0.7303 (0.0690)	.97	0.0045 (0.0067)	.51
COVID-19 mortality per 10 000	0.0013 (0.0033)	.70	0.9741 (0.0490)	<.001	0.0142 (0.0063)	.02

Abbreviations: IV, instrumental variable; NA, not applicable; VHA, Veterans Health Administration.

^a^ Each model used 66 387 observations and station and month fixed effects.

The first-stage findings of the IV probit analysis illustrated the strength of our instrument, which was substantial and statistically significant. Specifically, the Cragg–Donald Wald *F* statistic was 116.4, and the Kleibergen-Paap Wald *F* statistic was 40.4. Given that the critical value of the Stock-Yogo weak instrument test for 10% bias is 16.4, these *F* statistics indicated that the instrument was sufficiently strong to avoid weak instrument bias.

In the second stage, the coefficient associated with the independent variable of interest (percentage of virtual care) was negative and statistically significant (coefficient, −0.0161 [robust standard error, 0.0049]; *P* = .001) (Table 2). The elasticity of SREs with respect to the proportion of virtual care visits was −2.5, which meant that a 1% increase in the probability of virtual mental health visits was associated with a 2.5% decrease in SREs (0.07 percentage points). The IV results were larger in magnitude than those obtained through single-equation probit regression analysis. Marginal effects of the second stage results are reported in eAppendix 7 in Supplement 1.

Sensitivity analyses strengthened the validity of our primary models. Introducing a 1-month lead time for the SRE outcome variable in the model yielded statistically significant results (coefficient, −0.0179 [robust standard error, 0.0044]; *P* < .001) with an elasticity of −3.2. Similarly, the model that excluded data from early pandemic months was statistically significant (coefficient, −0.0178 [robust standard error, 0.0054]; *P* = .001) with an elasticity of −3.4. These outcomes, showing slightly larger elasticities compared with our primary model, suggest that our primary findings were on the conservative side. Full results of the sensitivity analyses can be found in eAppendices 8 and 9 in Supplement 1.

## Discussion

This national cohort study, using a quasi-experimental design, of virtual mental health use at the VHA between March 2020 and December 2021 found that an increase in the percentage of virtual mental health visits relative to the total visits was associated with a statistically significant decrease in SREs. This finding suggests that virtual mental health services may be protective in terms of suicide-related outcomes, corroborating previous descriptive research that reported beneficial impacts from the heightened uptake of telehealth services at the VHA.^30^

Our IV probit results were larger in magnitude compared with the findings obtained through a simple probit regression analysis. This result implies that the simple probit models may be underestimating the true effect. For instance, individuals at greater risk of suicide could be more inclined to use virtual mental health care, or there may be other unseen elements influencing both the propensity to opt for virtual care and the suicide risk. The consistency and statistical significance of the negative coefficients across both models indicate a reliable association between the variables studied.

There are several potential explanations for how the rise in virtual care use may contribute to a decrease in SREs. One mechanism could be the increased accessibility provided by telehealth.^47^ From the patient’s perspective, telehealth can cut down on travel time, enhance convenience, and lower or remove the costs linked to clinic visits.^48^ Another potential mechanism could be that some individuals might find virtual mental health care more comfortable. Research indicates that telehealth offers patients a sense of shared social connection with their physicians and that being in one’s home environment can enhance psychological safety.^49,50,51^ Furthermore, a significant advantage of telemental health is its potential to alleviate the stigma concerns often associated with seeking in-person care.^52^

Nevertheless, it is crucial to recognize that virtual mental health care does not serve all veterans uniformly. Qualitative research has noted that certain veterans may experience discomfort or have privacy worries when engaging with telehealth.^53^ Additional obstacles may stem from technological limitations, such as unreliable internet connections or lack of necessary technology.^54^ Studies also reveal significant disparities in access to telehealth video services based on race and socioeconomic status.^55^ Therefore, maintaining both virtual and in-person care options is essential to address these varied needs and barriers.^52^

### Limitations

This evaluation has several limitations. First, it is possible that the zip codes for veterans sourced from the DoD data may not be precise or may represent addresses where the veterans no longer live, such as their parents’ homes. However, this was not a major concern because only 7% of the entire sample was identified using DoD data. Second, the findings may not be generalizable to nonveteran populations because veterans differ notably from the wider US population in terms of demographics and comorbidities, as well as experiences, such as exposure to combat.^56^ Therefore, the implications of our findings should be considered carefully, as the unique experiences of veterans may significantly influence the outcomes. Future research should aim to include samples that are more representative of the general US population or focus on different subgroups within the general population to validate these findings in other contexts. However, given that the VHA operates one of the largest mental telehealth networks in the world, our conclusions still have significant implications for global mental telehealth guidelines and practices. Third, there remains a possibility that unmeasured confounders may influence both broadband access and the outcome of SREs. This underlying issue could potentially distort the causal interpretation of our findings. However, this concern is mitigated by the fact that simple probit results align in the same direction as the IV results, suggesting consistency in the observed associations. Fourth, these findings may not be generalizable to all veteran populations, as recently separated veterans may face distinct challenges, such as less familiarity with VHA services and greater barriers to accessing mental health care, compared with veterans with established VHA care. Fifth, the results may not be generalizable to 2019 or earlier given how the landscape of virtual care has changed as a result of the COVID-19 pandemic. Sixth, these results apply only to specialty mental health care provided by psychiatrists, psychologists, and social workers. Finally, specific subsets of the population may experience different results. Future research may benefit from conducting analyses that are stratified by different socioeconomic and racial and ethnic demographics, as well as various comorbidities. Extending the evaluation time frame beyond 2021 could provide valuable insights into whether the observed associations persist during the latter stages of the COVID-19–pandemic era and beyond. Despite these limitations, to our knowledge, this was the first quasi-experimental evaluation to explore the association between virtual mental health care use and SREs.

## Conclusions

This national cohort study using a retrospective quasi-experimental design found that increased use of virtual mental health services at the VHA was associated with a significant reduction in SREs among veterans. Our results support continued expansion of mental telehealth within the VHA. Future evaluations should seek to confirm these findings among different demographic groups and extend beyond the COVID-19–pandemic era.

## Notes

Supplement 1. **eAppendix 1.** Sample matching process **eAppendix 2.** Sample missingness and inclusion/exclusion process **eAppendix 3.** Non-fatal suicide attempt and intentional self-harm ICD-10 codes used **eAppendix 4.** Falsification test **eAppendix 5.** Heckman correction **eAppendix 6.** Average virtual care use by demographic subgroups **eAppendix 7.** Probit and IV Probit second stage marginal effects **eAppendix 8.** Sensitivity analysis: one month lead in suicide-related events **eAppendix 9.** Table 4. Sensitivity analysis: exclusion of March, April, and May 2020 data

Supplement 2.
Data Sharing Statement
