# Supplementary material for: Virtual Mental Health Care and Suicide-Related Events
Source: JAMA Netw Open. 2024 Nov 5;7(11):e2443054. doi: 10.1001/jamanetworkopen.2024.43054 (PMC11539012; doi:10.1001/jamanetworkopen.2024.43054)
Supplement: Supplement 1. — eAppendix 1. Sample matching process eAppendix 2. Sample missingness and inclusion/exclusion process eAppendix 3. Non-fatal suicide attempt and intentional self-harm ICD-10 codes used eAppendix 4. Falsification test eAppendix 5. Heckman correction eAppendix 6. Average virtual care use by demographic subgroups eAppendix 7. Probit and IV Probit second stage marginal effects eAppendix 8. Sensitivity analysis: one month lead in suicide-related events eAppendix 9. Table 4. Sensitivity analysis: exclusion of March, April, and May 2020 data [file jamanetwopen-e2443054-s001.pdf]

Supplementary Online Content

Tenso K, Strombotne K, Garrido MM, Lum J, Pizer S. Comparative effectiveness of virtual mental health care on suicide-related events. *JAMA Netw Open*. 2024;7(11):e2443054. doi:10.1001/jamanetworkopen.2024.43054

eAppendix 1. Sample matching process
eAppendix 2. Sample missingness and inclusion/exclusion process
eAppendix 3. Non-fatal suicide attempt and intentional self-harm ICD-10 codes used
eAppendix 4. Falsification test
eAppendix 5. Heckman correction
eAppendix 6. Average virtual care use by demographic subgroups
eAppendix 7. Probit and IVProbit second stage marginal effects
eAppendix 8. Sensitivity analysis: one month lead in suicide-related events
eAppendix 9. Table 4. Sensitivity analysis: exclusion of March, April, and May 2020 data

This supplementary material has been provided by the authors to give readers additional information about their work.

© 2024 Tenso K et al. *JAMA Network Open*.

eAppendix 1. Sample matching process

We used a three-step process to match each Veteran with their nearest primary care facility. In the first step, we matched each individual-month-year with the VA's Planning Systems Support Group (PSSG) annual data, which contains information on enrolled Veterans' addresses and uses geocoding to estimate the closest primary care facility for each Veteran, along with the driving distance and travel time to reach that facility. In the second step, we attempted to fill in missing observations by using the PSSG file from the following year. It's important to note that depending on the separation date, some Veterans might not have appeared in the PSSG file immediately. For the third step, we matched Veterans to their closest primary care facility based on Department of Defense (DoD) zip codes. Due to data limitations, observations for the period from October to December 2021 were matched using data from the prior fiscal year's PSSG file. The results showed that 86% of the observations were successfully matched to a primary care facility during the first attempt, 6% during the second attempt, 7% during the third attempt, and 0.3% of the observations remained unmatched.

© 2024 Tenso K et al. *JAMA Network Open*.

eAppendix2. Sample missingness and inclusion/exclusion process

324,692 obs (34,286 Vets)

Recently separated Veterans with past behavioral health diagnoses

↓

320,749 obs (33,980 Vets)

Dropped observations with no SOE data

↓

320,733 obs (33,979 Vets)

Dropped observations unable to be matched to FCC data using FIPS

↓

320,439 obs (33,949 Vets)

Dropped observations with no Covid data

↓

319,775 obs (33,898 Vets)

Dropped observations that happen during or after Veteran's death month

↓

69,273 obs (16,894 Vets)

Dropped observations with no mental health utilization

↓

67,424 (16,465 Vets)

Dropped observations that were collinear or predicted failure perfectly in regression models

© 2024 Tenso K et al. *JAMA Network Open*.

eAppendix 3. Non-fatal suicide attempt and intentional self-harm ICD-10 codes used

ICD10Code	ICD10Description
T14.91	Suicide Attempt
T14.91XA	Suicide attempt, initial encounter
T14.91XD	Suicide attempt, subsequent encounter
T36.0X2A	Poisoning by penicillins, intentional self-harm, initial encounter
T36.0X2D	Poisoning by penicillins, intentional self-harm, subsequent encounter
T36.1X2A	Poisoning by cephalosporins and other beta-lactam antibiotics, intentional self-harm, initial encounter
T36.1X2D	Poisoning by cephalosporins and other beta-lactam antibiotics, intentional self-harm, subsequent encounter
T36.2X2A	Poisoning by chloramphenicol group, intentional self-harm, initial encounter
T36.2X2D	Poisoning by chloramphenicol group, intentional self-harm, subsequent encounter
T36.3X2A	Poisoning by macrolides, intentional self-harm, initial encounter
T36.3X2D	Poisoning by macrolides, intentional self-harm, subsequent encounter
T36.4X2A	Poisoning by tetracyclines, intentional self-harm, initial encounter
T36.4X2D	Poisoning by tetracyclines, intentional self-harm, subsequent encounter
T36.5X2A	Poisoning by aminoglycosides, intentional self-harm, initial encounter
T36.5X2D	Poisoning by aminoglycosides, intentional self-harm, subsequent encounter
T36.6X2A	Poisoning by rifampicins, intentional self-harm, initial encounter
T36.6X2D	Poisoning by rifampicins, intentional self-harm, subsequent encounter
T36.7X2A	Poisoning by antifungal antibiotics, systemically used, intentional self-harm, initial encounter
T36.7X2D	Poisoning by antifungal antibiotics, systemically used, intentional self-harm, subsequent encounter
T36.8X2A	Poisoning by other systemic antibiotics, intentional self-harm, initial encounter
T36.8X2D	Poisoning by other systemic antibiotics, intentional self-harm, subsequent encounter
T36.92XA	Poisoning by unspecified systemic antibiotic, intentional self-harm, initial encounter
T36.92XD	Poisoning by unspecified systemic antibiotic, intentional self-harm, subsequent encounter
T37.0X2A	Poisoning by sulfonamides, intentional self-harm, initial encounter
T37.0X2D	Poisoning by sulfonamides, intentional self-harm, subsequent encounter
T37.1X2A	Poisoning by antimycobacterial drugs, intentional self-harm, initial encounter
T37.1X2D	Poisoning by antimycobacterial drugs, intentional self-harm, subsequent encounter
T37.2X2A	Poisoning by antimalarials and drugs acting on other blood protozoa, intentional self-harm, initial encounter
T37.2X2D	Poisoning by antimalarials and drugs acting on other blood protozoa, intentional self-harm, subsequent encounter
T37.3X2A	Poisoning by other antiprotozoal drugs, intentional self-harm, initial encounter
T37.3X2D	Poisoning by other antiprotozoal drugs, intentional self-harm, subsequent encounter
T37.4X2A	Poisoning by anthelminthics, intentional self-harm, initial encounter
T37.4X2D	Poisoning by anthelminthics, intentional self-harm, subsequent encounter
T37.5X2A	Poisoning by antiviral drugs, intentional self-harm, initial encounter
T37.5X2D	Poisoning by antiviral drugs, intentional self-harm, subsequent encounter

© 2024 Tenso K et al. *JAMA Network Open*.

T37.8X2A	Poisoning by other specified systemic anti-infectives and antiparasitics, intentional self-harm, initial encounter
T37.8X2D	Poisoning by other specified systemic anti-infectives and antiparasitics, intentional self-harm, subsequent encounter
T37.92XA	Poisoning by unspecified systemic anti-infective and antiparasitics, intentional self-harm, initial encounter
T37.92XD	Poisoning by unspecified systemic anti-infective and antiparasitics, intentional self-harm, subsequent encounter
T38.0X2A	Poisoning by glucocorticoids and synthetic analogues, intentional self-harm, initial encounter
T38.0X2D	Poisoning by glucocorticoids and synthetic analogues, intentional self-harm, subsequent encounter
T38.1X2A	Poisoning by thyroid hormones and substitutes, intentional self-harm, initial encounter
T38.1X2D	Poisoning by thyroid hormones and substitutes, intentional self-harm, subsequent encounter
T38.2X2A	Poisoning by antithyroid drugs, intentional self-harm, initial encounter
T38.2X2D	Poisoning by antithyroid drugs, intentional self-harm, subsequent encounter
T38.3X2A	Poisoning by insulin and oral hypoglycemic [antidiabetic] drugs, intentional self-harm, initial encounter
T38.3X2D	Poisoning by insulin and oral hypoglycemic [antidiabetic] drugs, intentional self-harm, subsequent encounter
T38.4X2A	Poisoning by oral contraceptives, intentional self-harm, initial encounter
T38.4X2D	Poisoning by oral contraceptives, intentional self-harm, subsequent encounter
T38.5X2A	Poisoning by other estrogens and progestogens, intentional self-harm, initial encounter
T38.5X2D	Poisoning by other estrogens and progestogens, intentional self-harm, subsequent encounter
T38.6X2A	Poisoning by antigonadotrophins, antiestrogens, antiandrogens, not elsewhere classified, intentional self-harm, initial encounter
T38.6X2D	Poisoning by antigonadotrophins, antiestrogens, antiandrogens, not elsewhere classified, intentional self-harm, subsequent encounter
T38.7X2A	Poisoning by androgens and anabolic congeners, intentional self-harm, initial encounter
T38.7X2D	Poisoning by androgens and anabolic congeners, intentional self-harm, subsequent encounter
T38.802A	Poisoning by unspecified hormones and synthetic substitutes, intentional self-harm, initial encounter
T38.802D	Poisoning by unspecified hormones and synthetic substitutes, intentional self-harm, subsequent encounter
T38.812A	Poisoning by anterior pituitary [adenohypophyseal] hormones, intentional self-harm, initial encounter
T38.812D	Poisoning by anterior pituitary [adenohypophyseal] hormones, intentional self-harm, subsequent encounter
T38.892A	Poisoning by other hormones and synthetic substitutes, intentional self-harm, initial encounter
T38.892D	Poisoning by other hormones and synthetic substitutes, intentional self-harm, subsequent encounter
T38.902A	Poisoning by unspecified hormone antagonists, intentional self-harm, initial encounter
T38.902D	Poisoning by unspecified hormone antagonists, intentional self-harm, subsequent encounter
T38.992A	Poisoning by other hormone antagonists, intentional self-harm, initial encounter
T38.992D	Poisoning by other hormone antagonists, intentional self-harm, subsequent encounter
T39.012A	Poisoning by aspirin, intentional self-harm, initial encounter
T39.012D	Poisoning by aspirin, intentional self-harm, subsequent encounter
T39.092A	Poisoning by salicylates, intentional self-harm, initial encounter
T39.092D	Poisoning by salicylates, intentional self-harm, subsequent encounter

© 2024 Tenso K et al. *JAMA Network Open*.

T39.1X2A	Poisoning by 4-Aminophenol derivatives, intentional self-harm, initial encounter
T39.1X2D	Poisoning by 4-Aminophenol derivatives, intentional self-harm, subsequent encounter
T39.2X2A	Poisoning by pyrazolone derivatives, intentional self-harm, initial encounter
T39.2X2D	Poisoning by pyrazolone derivatives, intentional self-harm, subsequent encounter
T39.312A	Poisoning by propionic acid derivatives, intentional self-harm, initial encounter
T39.312D	Poisoning by propionic acid derivatives, intentional self-harm, subsequent encounter
T39.392A	Poisoning by other nonsteroidal anti-inflammatory drugs [NSAID], intentional self-harm, initial encounter
T39.392D	Poisoning by other nonsteroidal anti-inflammatory drugs [NSAID], intentional self-harm, subsequent encounter
T39.4X2A	Poisoning by antirheumatics, not elsewhere classified, intentional self-harm, initial encounter
T39.4X2D	Poisoning by antirheumatics, not elsewhere classified, intentional self-harm, subsequent encounter
T39.8X2A	Poisoning by other nonopioid analgesics and antipyretics, not elsewhere classified, intentional self-harm, initial encounter
T39.8X2D	Poisoning by other nonopioid analgesics and antipyretics, not elsewhere classified, intentional self-harm, subsequent encounter
T39.92XA	Poisoning by unspecified nonopioid analgesic, antipyretic and antirheumatic, intentional self-harm, initial encounter
T39.92XD	Poisoning by unspecified nonopioid analgesic, antipyretic and antirheumatic, intentional self-harm, subsequent encounter
T40.0X2A	Poisoning by opium, intentional self-harm, initial encounter
T40.0X2D	Poisoning by opium, intentional self-harm, subsequent encounter
T40.1X2A	Poisoning by heroin, intentional self-harm, initial encounter
T40.1X2D	Poisoning by heroin, intentional self-harm, subsequent encounter
T40.2X2A	Poisoning by other opioids, intentional self-harm, initial encounter
T40.2X2D	Poisoning by other opioids, intentional self-harm, subsequent encounter
T40.3X2A	Poisoning by methadone, intentional self-harm, initial encounter
T40.3X2D	Poisoning by methadone, intentional self-harm, subsequent encounter
T40.4X2A	Poisoning by other synthetic narcotics, intentional self-harm, initial encounter
T40.4X2D	Poisoning by other synthetic narcotics, intentional self-harm, subsequent encounter
T40.5X2A	Poisoning by cocaine, intentional self-harm, initial encounter
T40.5X2D	Poisoning by cocaine, intentional self-harm, subsequent encounter
T40.602A	Poisoning by unspecified narcotics, intentional self-harm, initial encounter
T40.602D	Poisoning by unspecified narcotics, intentional self-harm, subsequent encounter
T40.692A	Poisoning by other narcotics, intentional self-harm, initial encounter
T40.692D	Poisoning by other narcotics, intentional self-harm, subsequent encounter
T40.7X2A	Poisoning by cannabis (derivatives), intentional self-harm, initial encounter
T40.7X2D	Poisoning by cannabis (derivatives), intentional self-harm, subsequent encounter
T40.8X2A	Poisoning by lysergide [LSD], intentional self-harm, initial encounter
T40.8X2D	Poisoning by lysergide [LSD], intentional self-harm, subsequent encounter
T40.902A	Poisoning by unspecified psychodysleptics [hallucinogens], intentional self-harm, initial encounter
T40.902D	Poisoning by unspecified psychodysleptics [hallucinogens], intentional self-harm, subsequent encounter
T40.992A	Poisoning by other psychodysleptics [hallucinogens], intentional self-harm, initial encounter

© 2024 Tenso K et al. *JAMA Network Open*.

T40.992D	Poisoning by other psychodysleptics [hallucinogens], intentional self-harm, subsequent encounter
T41.0X2A	Poisoning by inhaled anesthetics, intentional self-harm, initial encounter
T41.0X2D	Poisoning by inhaled anesthetics, intentional self-harm, subsequent encounter
T41.1X2A	Poisoning by intravenous anesthetics, intentional self-harm, initial encounter
T41.1X2D	Poisoning by intravenous anesthetics, intentional self-harm, subsequent encounter
T41.202A	Poisoning by unspecified general anesthetics, intentional self-harm, initial encounter
T41.202D	Poisoning by unspecified general anesthetics, intentional self-harm, subsequent encounter
T41.292A	Poisoning by other general anesthetics, intentional self-harm, initial encounter
T41.292D	Poisoning by other general anesthetics, intentional self-harm, subsequent encounter
T41.3X2A	Poisoning by local anesthetics, intentional self-harm, initial encounter
T41.3X2D	Poisoning by local anesthetics, intentional self-harm, subsequent encounter
T41.42XA	Poisoning by unspecified anesthetic, intentional self-harm, initial encounter
T41.42XD	Poisoning by unspecified anesthetic, intentional self-harm, subsequent encounter
T41.5X2A	Poisoning by therapeutic gases, intentional self-harm, initial encounter
T41.5X2D	Poisoning by therapeutic gases, intentional self-harm, subsequent encounter
T42.0X2A	Poisoning by hydantoin derivatives, intentional self-harm, initial encounter
T42.0X2D	Poisoning by hydantoin derivatives, intentional self-harm, subsequent encounter
T42.1X2A	Poisoning by iminostilbenes, intentional self-harm, initial encounter
T42.1X2D	Poisoning by iminostilbenes, intentional self-harm, subsequent encounter
T42.2X2A	Poisoning by succinimides and oxazolidinediones, intentional self-harm, initial encounter
T42.2X2D	Poisoning by succinimides and oxazolidinediones, intentional self-harm, subsequent encounter
T42.3X2A	Poisoning by barbiturates, intentional self-harm, initial encounter
T42.3X2D	Poisoning by barbiturates, intentional self-harm, subsequent encounter
T42.4X2A	Poisoning by benzodiazepines, intentional self-harm, initial encounter
T42.4X2D	Poisoning by benzodiazepines, intentional self-harm, subsequent encounter
T42.5X2A	Poisoning by mixed antiepileptics, intentional self-harm, initial encounter
T42.5X2D	Poisoning by mixed antiepileptics, intentional self-harm, subsequent encounter
T42.6X2A	Poisoning by other antiepileptic and sedative-hypnotic drugs, intentional self-harm, initial encounter
T42.6X2D	Poisoning by other antiepileptic and sedative-hypnotic drugs, intentional self-harm, subsequent encounter
T42.72XA	Poisoning by unspecified antiepileptic and sedative-hypnotic drugs, intentional self-harm, initial encounter
T42.72XD	Poisoning by unspecified antiepileptic and sedative-hypnotic drugs, intentional self-harm, subsequent encounter
T42.8X2A	Poisoning by antiparkinsonism drugs and other central muscle-tone depressants, intentional self-harm, initial encounter
T42.8X2D	Poisoning by antiparkinsonism drugs and other central muscle-tone depressants, intentional self-harm, subsequent encounter
T43.012A	Poisoning by tricyclic antidepressants, intentional self-harm, initial encounter
T43.012D	Poisoning by tricyclic antidepressants, intentional self-harm, subsequent encounter
T43.022A	Poisoning by tetracyclic antidepressants, intentional self-harm, initial encounter
T43.022D	Poisoning by tetracyclic antidepressants, intentional self-harm, subsequent encounter
T43.1X2A	Poisoning by monoamine-oxidase-inhibitor antidepressants, intentional self-harm, initial encounter

© 2024 Tenso K et al. *JAMA Network Open*.

T43.1X2D	Poisoning by monoamine-oxidase-inhibitor antidepressants, intentional self-harm, subsequent encounter
T43.202A	Poisoning by unspecified antidepressants, intentional self-harm, initial encounter
T43.202D	Poisoning by unspecified antidepressants, intentional self-harm, subsequent encounter
T43.212A	Poisoning by selective serotonin and norepinephrine reuptake inhibitors, intentional self-harm, initial encounter
T43.212D	Poisoning by selective serotonin and norepinephrine reuptake inhibitors, intentional self-harm, subsequent encounter
T43.222A	Poisoning by selective serotonin reuptake inhibitors, intentional self-harm, initial encounter
T43.222D	Poisoning by selective serotonin reuptake inhibitors, intentional self-harm, subsequent encounter
T43.292A	Poisoning by other antidepressants, intentional self-harm, initial encounter
T43.292D	Poisoning by other antidepressants, intentional self-harm, subsequent encounter
T43.3X2A	Poisoning by phenothiazine antipsychotics and neuroleptics, intentional self-harm, initial encounter
T43.3X2D	Poisoning by phenothiazine antipsychotics and neuroleptics, intentional self-harm, subsequent encounter
T43.4X2A	Poisoning by butyrophenone and thiothixene neuroleptics, intentional self-harm, initial encounter
T43.4X2D	Poisoning by butyrophenone and thiothixene neuroleptics, intentional self-harm, subsequent encounter
T43.502A	Poisoning by unspecified antipsychotics and neuroleptics, intentional self-harm, initial encounter
T43.502D	Poisoning by unspecified antipsychotics and neuroleptics, intentional self-harm, subsequent encounter
T43.592A	Poisoning by other antipsychotics and neuroleptics, intentional self-harm, initial encounter
T43.592D	Poisoning by other antipsychotics and neuroleptics, intentional self-harm, subsequent encounter
T43.602A	Poisoning by unspecified psychostimulants, intentional self-harm, initial encounter
T43.602D	Poisoning by unspecified psychostimulants, intentional self-harm, subsequent encounter
T43.612A	Poisoning by caffeine, intentional self-harm, initial encounter
T43.612D	Poisoning by caffeine, intentional self-harm, subsequent encounter
T43.622A	Poisoning by amphetamines, intentional self-harm, initial encounter
T43.622D	Poisoning by amphetamines, intentional self-harm, subsequent encounter
T43.632A	Poisoning by methylphenidate, intentional self-harm, initial encounter
T43.632D	Poisoning by methylphenidate, intentional self-harm, subsequent encounter
T43.642A	Poisoning by ecstasy, intentional self-harm, initial encounter
T43.642D	Poisoning by ecstasy, intentional self-harm, subsequent encounter
T43.692A	Poisoning by other psychostimulants, intentional self-harm, initial encounter
T43.692D	Poisoning by other psychostimulants, intentional self-harm, subsequent encounter
T43.8X2A	Poisoning by other psychotropic drugs, intentional self-harm, initial encounter
T43.8X2D	Poisoning by other psychotropic drugs, intentional self-harm, subsequent encounter
T43.92XA	Poisoning by unspecified psychotropic drug, intentional self-harm, initial encounter
T43.92XD	Poisoning by unspecified psychotropic drug, intentional self-harm, subsequent encounter
T44.0X2A	Poisoning by anticholinesterase agents, intentional self-harm, initial encounter
T44.0X2D	Poisoning by anticholinesterase agents, intentional self-harm, subsequent encounter
T44.1X2A	Poisoning by other parasympathomimetics [cholinergics], intentional self-harm, initial encounter
T44.1X2D	Poisoning by other parasympathomimetics [cholinergics], intentional self-harm, subsequent encounter

© 2024 Tenso K et al. *JAMA Network Open*.

T44.2X2A	Poisoning by ganglionic blocking drugs, intentional self-harm, initial encounter
T44.2X2D	Poisoning by ganglionic blocking drugs, intentional self-harm, subsequent encounter
T44.3X2A	Poisoning by other parasympatholytics [anticholinergics and antimuscarinics] and spasmolytics, intentional self-harm, initial encounter
T44.3X2D	Poisoning by other parasympatholytics [anticholinergics and antimuscarinics] and spasmolytics, intentional self-harm, subsequent encounter
T44.4X2A	Poisoning by predominantly alpha-adrenoreceptor agonists, intentional self-harm, initial encounter
T44.4X2D	Poisoning by predominantly alpha-adrenoreceptor agonists, intentional self-harm, subsequent encounter
T44.5X2A	Poisoning by predominantly beta-adrenoreceptor agonists, intentional self-harm, initial encounter
T44.5X2D	Poisoning by predominantly beta-adrenoreceptor agonists, intentional self-harm, subsequent encounter
T44.6X2A	Poisoning by alpha-adrenoreceptor antagonists, intentional self-harm, initial encounter
T44.6X2D	Poisoning by alpha-adrenoreceptor antagonists, intentional self-harm, subsequent encounter
T44.7X2A	Poisoning by beta-adrenoreceptor antagonists, intentional self-harm, initial encounter
T44.7X2D	Poisoning by beta-adrenoreceptor antagonists, intentional self-harm, subsequent encounter
T44.8X2A	Poisoning by centrally-acting and adrenergic-neuron-blocking agents, intentional self-harm, initial encounter
T44.8X2D	Poisoning by centrally-acting and adrenergic-neuron-blocking agents, intentional self-harm, subsequent encounter
T44.902A	Poisoning by unspecified drugs primarily affecting the autonomic nervous system, intentional self-harm, initial encounter
T44.902D	Poisoning by unspecified drugs primarily affecting the autonomic nervous system, intentional self-harm, subsequent encounter
T44.992A	Poisoning by other drug primarily affecting the autonomic nervous system, intentional self-harm, initial encounter
T44.992D	Poisoning by other drug primarily affecting the autonomic nervous system, intentional self-harm, subsequent encounter
T45.0X2A	Poisoning by antiallergic and antiemetic drugs, intentional self-harm, initial encounter
T45.0X2D	Poisoning by antiallergic and antiemetic drugs, intentional self-harm, subsequent encounter
T45.1X2A	Poisoning by antineoplastic and immunosuppressive drugs, intentional self-harm, initial encounter
T45.1X2D	Poisoning by antineoplastic and immunosuppressive drugs, intentional self-harm, subsequent encounter
T45.2X2A	Poisoning by vitamins, intentional self-harm, initial encounter
T45.2X2D	Poisoning by vitamins, intentional self-harm, subsequent encounter
T45.3X2A	Poisoning by enzymes, intentional self-harm, initial encounter
T45.3X2D	Poisoning by enzymes, intentional self-harm, subsequent encounter
T45.4X2A	Poisoning by iron and its compounds, intentional self-harm, initial encounter
T45.4X2D	Poisoning by iron and its compounds, intentional self-harm, subsequent encounter
T45.512A	Poisoning by anticoagulants, intentional self-harm, initial encounter
T45.512D	Poisoning by anticoagulants, intentional self-harm, subsequent encounter
T45.522A	Poisoning by antithrombotic drugs, intentional self-harm, initial encounter
T45.522D	Poisoning by antithrombotic drugs, intentional self-harm, subsequent encounter
T45.602A	Poisoning by unspecified fibrinolysis-affecting drugs, intentional self-harm, initial encounter
T45.602D	Poisoning by unspecified fibrinolysis-affecting drugs, intentional self-harm, subsequent encounter
T45.612A	Poisoning by thrombolytic drug, intentional self-harm, initial encounter

© 2024 Tenso K et al. *JAMA Network Open*.

T45.612D	Poisoning by thrombolytic drug, intentional self-harm, subsequent encounter
T45.622A	Poisoning by hemostatic drug, intentional self-harm, initial encounter
T45.622D	Poisoning by hemostatic drug, intentional self-harm, subsequent encounter
T45.692A	Poisoning by other fibrinolysis-affecting drugs, intentional self-harm, initial encounter
T45.692D	Poisoning by other fibrinolysis-affecting drugs, intentional self-harm, subsequent encounter
T45.7X2A	Poisoning by anticoagulant antagonists, vitamin K and other coagulants, intentional self-harm, initial encounter
T45.7X2D	Poisoning by anticoagulant antagonists, vitamin K and other coagulants, intentional self-harm, subsequent encounter
T45.8X2A	Poisoning by other primarily systemic and hematological agents, intentional self-harm, initial encounter
T45.8X2D	Poisoning by other primarily systemic and hematological agents, intentional self-harm, subsequent encounter
T45.92XA	Poisoning by unspecified primarily systemic and hematological agent, intentional self-harm, initial encounter
T45.92XD	Poisoning by unspecified primarily systemic and hematological agent, intentional self-harm, subsequent encounter
T46.0X2A	Poisoning by cardiac-stimulant glycosides and drugs of similar action, intentional self-harm, initial encounter
T46.0X2D	Poisoning by cardiac-stimulant glycosides and drugs of similar action, intentional self-harm, subsequent encounter
T46.1X2A	Poisoning by calcium-channel blockers, intentional self-harm, initial encounter
T46.1X2D	Poisoning by calcium-channel blockers, intentional self-harm, subsequent encounter
T46.2X2A	Poisoning by other antidysrhythmic drugs, intentional self-harm, initial encounter
T46.2X2D	Poisoning by other antidysrhythmic drugs, intentional self-harm, subsequent encounter
T46.3X2A	Poisoning by coronary vasodilators, intentional self-harm, initial encounter
T46.3X2D	Poisoning by coronary vasodilators, intentional self-harm, subsequent encounter
T46.4X2A	Poisoning by angiotensin-converting-enzyme inhibitors, intentional self-harm, initial encounter
T46.4X2D	Poisoning by angiotensin-converting-enzyme inhibitors, intentional self-harm, subsequent encounter
T46.5X2A	Poisoning by other antihypertensive drugs, intentional self-harm, initial encounter
T46.5X2D	Poisoning by other antihypertensive drugs, intentional self-harm, subsequent encounter
T46.6X2A	Poisoning by antihyperlipidemic and antiarteriosclerotic drugs, intentional self-harm, initial encounter
T46.6X2D	Poisoning by antihyperlipidemic and antiarteriosclerotic drugs, intentional self-harm, subsequent encounter
T46.7X2A	Poisoning by peripheral vasodilators, intentional self-harm, initial encounter
T46.7X2D	Poisoning by peripheral vasodilators, intentional self-harm, subsequent encounter
T46.8X2A	Poisoning by antivaricose drugs, including sclerosing agents, intentional self-harm, initial encounter
T46.8X2D	Poisoning by antivaricose drugs, including sclerosing agents, intentional self-harm, subsequent encounter
T46.902A	Poisoning by unspecified agents primarily affecting the cardiovascular system, intentional self-harm, initial encounter
T46.902D	Poisoning by unspecified agents primarily affecting the cardiovascular system, intentional self-harm, subsequent encounter
T46.992A	Poisoning by other agents primarily affecting the cardiovascular system, intentional self-harm, initial encounter
T46.992D	Poisoning by other agents primarily affecting the cardiovascular system, intentional self-harm, subsequent encounter

© 2024 Tenso K et al. *JAMA Network Open*.

T47.0X2A	Poisoning by histamine H2-receptor blockers, intentional self-harm, initial encounter
T47.0X2D	Poisoning by histamine H2-receptor blockers, intentional self-harm, subsequent encounter
T47.1X2A	Poisoning by other antacids and anti-gastric-secretion drugs, intentional self-harm, initial encounter
T47.1X2D	Poisoning by other antacids and anti-gastric-secretion drugs, intentional self-harm, subsequent encounter
T47.2X2A	Poisoning by stimulant laxatives, intentional self-harm, initial encounter
T47.2X2D	Poisoning by stimulant laxatives, intentional self-harm, subsequent encounter
T47.3X2A	Poisoning by saline and osmotic laxatives, intentional self-harm, initial encounter
T47.3X2D	Poisoning by saline and osmotic laxatives, intentional self-harm, subsequent encounter
T47.4X2A	Poisoning by other laxatives, intentional self-harm, initial encounter
T47.4X2D	Poisoning by other laxatives, intentional self-harm, subsequent encounter
T47.5X2A	Poisoning by digestants, intentional self-harm, initial encounter
T47.5X2D	Poisoning by digestants, intentional self-harm, subsequent encounter
T47.6X2A	Poisoning by antidiarrheal drugs, intentional self-harm, initial encounter
T47.6X2D	Poisoning by antidiarrheal drugs, intentional self-harm, subsequent encounter
T47.7X2A	Poisoning by emetics, intentional self-harm, initial encounter
T47.7X2D	Poisoning by emetics, intentional self-harm, subsequent encounter
T47.8X2A	Poisoning by other agents primarily affecting gastrointestinal system, intentional self-harm, initial encounter
T47.8X2D	Poisoning by other agents primarily affecting gastrointestinal system, intentional self-harm, subsequent encounter
T47.92XA	Poisoning by unspecified agents primarily affecting the gastrointestinal system, intentional self-harm, initial encounter
T47.92XD	Poisoning by unspecified agents primarily affecting the gastrointestinal system, intentional self-harm, subsequent encounter
T48.0X2A	Poisoning by oxytocic drugs, intentional self-harm, initial encounter
T48.0X2D	Poisoning by oxytocic drugs, intentional self-harm, subsequent encounter
T48.1X2A	Poisoning by skeletal muscle relaxants [neuromuscular blocking agents], intentional self-harm, initial encounter
T48.1X2D	Poisoning by skeletal muscle relaxants [neuromuscular blocking agents], intentional self-harm, subsequent encounter
T48.202A	Poisoning by unspecified drugs acting on muscles, intentional self-harm, initial encounter
T48.202D	Poisoning by unspecified drugs acting on muscles, intentional self-harm, subsequent encounter
T48.292A	Poisoning by other drugs acting on muscles, intentional self-harm, initial encounter
T48.292D	Poisoning by other drugs acting on muscles, intentional self-harm, subsequent encounter
T48.3X2A	Poisoning by antitussives, intentional self-harm, initial encounter
T48.3X2D	Poisoning by antitussives, intentional self-harm, subsequent encounter
T48.4X2A	Poisoning by expectorants, intentional self-harm, initial encounter
T48.4X2D	Poisoning by expectorants, intentional self-harm, subsequent encounter
T48.5X2A	Poisoning by other anti-common-cold drugs, intentional self-harm, initial encounter
T48.5X2D	Poisoning by other anti-common-cold drugs, intentional self-harm, subsequent encounter
T48.6X2A	Poisoning by antiasthmatics, intentional self-harm, initial encounter
T48.6X2D	Poisoning by antiasthmatics, intentional self-harm, subsequent encounter
T48.902A	Poisoning by unspecified agents primarily acting on the respiratory system, intentional self-harm, initial encounter

© 2024 Tenso K et al. *JAMA Network Open*.

T48.902D	Poisoning by unspecified agents primarily acting on the respiratory system, intentional self-harm, subsequent encounter
T48.992A	Poisoning by other agents primarily acting on the respiratory system, intentional self-harm, initial encounter
T48.992D	Poisoning by other agents primarily acting on the respiratory system, intentional self-harm, subsequent encounter
T49.0X2A	Poisoning by local antifungal, anti-infective and anti-inflammatory drugs, intentional self-harm, initial encounter
T49.0X2D	Poisoning by local antifungal, anti-infective and anti-inflammatory drugs, intentional self-harm, subsequent encounter
T49.1X2A	Poisoning by antipruritics, intentional self-harm, initial encounter
T49.1X2D	Poisoning by antipruritics, intentional self-harm, subsequent encounter
T49.2X2A	Poisoning by local astringents and local detergents, intentional self-harm, initial encounter
T49.2X2D	Poisoning by local astringents and local detergents, intentional self-harm, subsequent encounter
T49.3X2A	Poisoning by emollients, demulcents and protectants, intentional self-harm, initial encounter
T49.3X2D	Poisoning by emollients, demulcents and protectants, intentional self-harm, subsequent encounter
T49.4X2A	Poisoning by keratolytics, keratoplastics, and other hair treatment drugs and preparations, intentional self-harm, initial encounter
T49.4X2D	Poisoning by keratolytics, keratoplastics, and other hair treatment drugs and preparations, intentional self-harm, subsequent encounter
T49.5X2A	Poisoning by ophthalmological drugs and preparations, intentional self-harm, initial encounter
T49.5X2D	Poisoning by ophthalmological drugs and preparations, intentional self-harm, subsequent encounter
T49.6X2A	Poisoning by otorhinolaryngological drugs and preparations, intentional self-harm, initial encounter
T49.6X2D	Poisoning by otorhinolaryngological drugs and preparations, intentional self-harm, subsequent encounter
T49.7X2A	Poisoning by dental drugs, topically applied, intentional self-harm, initial encounter
T49.7X2D	Poisoning by dental drugs, topically applied, intentional self-harm, subsequent encounter
T49.8X2A	Poisoning by other topical agents, intentional self-harm, initial encounter
T49.8X2D	Poisoning by other topical agents, intentional self-harm, subsequent encounter
T49.92XA	Poisoning by unspecified topical agent, intentional self-harm, initial encounter
T49.92XD	Poisoning by unspecified topical agent, intentional self-harm, subsequent encounter
T50.0X2A	Poisoning by mineralocorticoids and their antagonists, intentional self-harm, initial encounter
T50.0X2D	Poisoning by mineralocorticoids and their antagonists, intentional self-harm, subsequent encounter
T50.1X2A	Poisoning by loop [high-ceiling] diuretics, intentional self-harm, initial encounter
T50.1X2D	Poisoning by loop [high-ceiling] diuretics, intentional self-harm, subsequent encounter
T50.2X2A	Poisoning by carbonic-anhydrase inhibitors, benzothiadiazides and other diuretics, intentional self-harm, initial encounter
T50.2X2D	Poisoning by carbonic-anhydrase inhibitors, benzothiadiazides and other diuretics, intentional self-harm, subsequent encounter
T50.3X2A	Poisoning by electrolytic, caloric and water-balance agents, intentional self-harm, initial encounter
T50.3X2D	Poisoning by electrolytic, caloric and water-balance agents, intentional self-harm, subsequent encounter
T50.4X2A	Poisoning by drugs affecting uric acid metabolism, intentional self-harm, initial encounter
T50.4X2D	Poisoning by drugs affecting uric acid metabolism, intentional self-harm, subsequent encounter
T50.5X2A	Poisoning by appetite depressants, intentional self-harm, initial encounter

© 2024 Tenso K et al. *JAMA Network Open*.

T50.5X2D	Poisoning by appetite depressants, intentional self-harm, subsequent encounter
T50.6X2A	Poisoning by antidotes and chelating agents, intentional self-harm, initial encounter
T50.6X2D	Poisoning by antidotes and chelating agents, intentional self-harm, subsequent encounter
T50.7X2A	Poisoning by analeptics and opioid receptor antagonists, intentional self-harm, initial encounter
T50.7X2D	Poisoning by analeptics and opioid receptor antagonists, intentional self-harm, subsequent encounter
T50.8X2A	Poisoning by diagnostic agents, intentional self-harm, initial encounter
T50.8X2D	Poisoning by diagnostic agents, intentional self-harm, subsequent encounter
T50.902A	Poisoning by unspecified drugs, medicaments and biological substances, intentional self-harm, initial encounter
T50.902D	Poisoning by unspecified drugs, medicaments and biological substances, intentional self-harm, subsequent encounter
T50.992A	Poisoning by other drugs, medicaments and biological substances, intentional self-harm, initial encounter
T50.992D	Poisoning by other drugs, medicaments and biological substances, intentional self-harm, subsequent encounter
T50.A12A	Poisoning by pertussis vaccine, including combinations with a pertussis component, intentional self-harm, initial encounter
T50.A12D	Poisoning by pertussis vaccine, including combinations with a pertussis component, intentional self-harm, subsequent encounter
T50.A22A	Poisoning by mixed bacterial vaccines without a pertussis component, intentional self-harm, initial encounter
T50.A22D	Poisoning by mixed bacterial vaccines without a pertussis component, intentional self-harm, subsequent encounter
T50.A92A	Poisoning by other bacterial vaccines, intentional self-harm, initial encounter
T50.A92D	Poisoning by other bacterial vaccines, intentional self-harm, subsequent encounter
T50.B12A	Poisoning by smallpox vaccines, intentional self-harm, initial encounter
T50.B12D	Poisoning by smallpox vaccines, intentional self-harm, subsequent encounter
T50.B92A	Poisoning by other viral vaccines, intentional self-harm, initial encounter
T50.B92D	Poisoning by other viral vaccines, intentional self-harm, subsequent encounter
T50.Z12A	Poisoning by immunoglobulin, intentional self-harm, initial encounter
T50.Z12D	Poisoning by immunoglobulin, intentional self-harm, subsequent encounter
T50.Z92A	Poisoning by other vaccines and biological substances, intentional self-harm, initial encounter
T50.Z92D	Poisoning by other vaccines and biological substances, intentional self-harm, subsequent encounter
T51.0X2A	Toxic effect of ethanol, intentional self-harm, initial encounter
T51.0X2D	Toxic effect of ethanol, intentional self-harm, subsequent encounter
T51.1X2A	Toxic effect of methanol, intentional self-harm, initial encounter
T51.1X2D	Toxic effect of methanol, intentional self-harm, subsequent encounter
T51.2X2A	Toxic effect of 2-Propanol, intentional self-harm, initial encounter
T51.2X2D	Toxic effect of 2-Propanol, intentional self-harm, subsequent encounter
T51.3X2A	Toxic effect of fusel oil, intentional self-harm, initial encounter
T51.3X2D	Toxic effect of fusel oil, intentional self-harm, subsequent encounter
T51.8X2A	Toxic effect of other alcohols, intentional self-harm, initial encounter
T51.8X2D	Toxic effect of other alcohols, intentional self-harm, subsequent encounter
T51.92XA	Toxic effect of unspecified alcohol, intentional self-harm, initial encounter

© 2024 Tenso K et al. *JAMA Network Open*.

T51.92XD	Toxic effect of unspecified alcohol, intentional self-harm, subsequent encounter
T52.0X2A	Toxic effect of petroleum products, intentional self-harm, initial encounter
T52.0X2D	Toxic effect of petroleum products, intentional self-harm, subsequent encounter
T52.1X2A	Toxic effect of benzene, intentional self-harm, initial encounter
T52.1X2D	Toxic effect of benzene, intentional self-harm, subsequent encounter
T52.2X2A	Toxic effect of homologues of benzene, intentional self-harm, initial encounter
T52.2X2D	Toxic effect of homologues of benzene, intentional self-harm, subsequent encounter
T52.3X2A	Toxic effect of glycols, intentional self-harm, initial encounter
T52.3X2D	Toxic effect of glycols, intentional self-harm, subsequent encounter
T52.4X2A	Toxic effect of ketones, intentional self-harm, initial encounter
T52.4X2D	Toxic effect of ketones, intentional self-harm, subsequent encounter
T52.8X2A	Toxic effect of other organic solvents, intentional self-harm, initial encounter
T52.8X2D	Toxic effect of other organic solvents, intentional self-harm, subsequent encounter
T52.92XA	Toxic effect of unspecified organic solvent, intentional self-harm, initial encounter
T52.92XD	Toxic effect of unspecified organic solvent, intentional self-harm, subsequent encounter
T53.0X2A	Toxic effect of carbon tetrachloride, intentional self-harm, initial encounter
T53.0X2D	Toxic effect of carbon tetrachloride, intentional self-harm, subsequent encounter
T53.1X2A	Toxic effect of chloroform, intentional self-harm, initial encounter
T53.1X2D	Toxic effect of chloroform, intentional self-harm, subsequent encounter
T53.2X2A	Toxic effect of trichloroethylene, intentional self-harm, initial encounter
T53.2X2D	Toxic effect of trichloroethylene, intentional self-harm, subsequent encounter
T53.3X2A	Toxic effect of tetrachloroethylene, intentional self-harm, initial encounter
T53.3X2D	Toxic effect of tetrachloroethylene, intentional self-harm, subsequent encounter
T53.4X2A	Toxic effect of dichloromethane, intentional self-harm, initial encounter
T53.4X2D	Toxic effect of dichloromethane, intentional self-harm, subsequent encounter
T53.5X2A	Toxic effect of chlorofluorocarbons, intentional self-harm, initial encounter
T53.5X2D	Toxic effect of chlorofluorocarbons, intentional self-harm, subsequent encounter
T53.6X2A	Toxic effect of other halogen derivatives of aliphatic hydrocarbons, intentional self-harm, initial encounter
T53.6X2D	Toxic effect of other halogen derivatives of aliphatic hydrocarbons, intentional self-harm, subsequent encounter
T53.7X2A	Toxic effect of other halogen derivatives of aromatic hydrocarbons, intentional self-harm, initial encounter
T53.7X2D	Toxic effect of other halogen derivatives of aromatic hydrocarbons, intentional self-harm, subsequent encounter
T53.92XA	Toxic effect of unspecified halogen derivatives of aliphatic and aromatic hydrocarbons, intentional self-harm, initial encounter
T53.92XD	Toxic effect of unspecified halogen derivatives of aliphatic and aromatic hydrocarbons, intentional self-harm, subsequent encounter
T54.0X2A	Toxic effect of phenol and phenol homologues, intentional self-harm, initial encounter
T54.0X2D	Toxic effect of phenol and phenol homologues, intentional self-harm, subsequent encounter
T54.1X2A	Toxic effect of other corrosive organic compounds, intentional self-harm, initial encounter
T54.1X2D	Toxic effect of other corrosive organic compounds, intentional self-harm, subsequent encounter
T54.2X2A	Toxic effect of corrosive acids and acid-like substances, intentional self-harm, initial encounter

© 2024 Tenso K et al. *JAMA Network Open*.

T54.2X2D	Toxic effect of corrosive acids and acid-like substances, intentional self-harm, subsequent encounter
T54.3X2A	Toxic effect of corrosive alkalis and alkali-like substances, intentional self-harm, initial encounter
T54.3X2D	Toxic effect of corrosive alkalis and alkali-like substances, intentional self-harm, subsequent encounter
T54.92XA	Toxic effect of unspecified corrosive substance, intentional self-harm, initial encounter
T54.92XD	Toxic effect of unspecified corrosive substance, intentional self-harm, subsequent encounter
T55.0X2A	Toxic effect of soaps, intentional self-harm, initial encounter
T55.0X2D	Toxic effect of soaps, intentional self-harm, subsequent encounter
T55.1X2A	Toxic effect of detergents, intentional self-harm, initial encounter
T55.1X2D	Toxic effect of detergents, intentional self-harm, subsequent encounter
T56.0X2A	Toxic effect of lead and its compounds, intentional self-harm, initial encounter
T56.0X2D	Toxic effect of lead and its compounds, intentional self-harm, subsequent encounter
T56.1X2A	Toxic effect of mercury and its compounds, intentional self-harm, initial encounter
T56.1X2D	Toxic effect of mercury and its compounds, intentional self-harm, subsequent encounter
T56.2X2A	Toxic effect of chromium and its compounds, intentional self-harm, initial encounter
T56.2X2D	Toxic effect of chromium and its compounds, intentional self-harm, subsequent encounter
T56.3X2A	Toxic effect of cadmium and its compounds, intentional self-harm, initial encounter
T56.3X2D	Toxic effect of cadmium and its compounds, intentional self-harm, subsequent encounter
T56.4X2A	Toxic effect of copper and its compounds, intentional self-harm, initial encounter
T56.4X2D	Toxic effect of copper and its compounds, intentional self-harm, subsequent encounter
T56.5X2A	Toxic effect of zinc and its compounds, intentional self-harm, initial encounter
T56.5X2D	Toxic effect of zinc and its compounds, intentional self-harm, subsequent encounter
T56.6X2A	Toxic effect of tin and its compounds, intentional self-harm, initial encounter
T56.6X2D	Toxic effect of tin and its compounds, intentional self-harm, subsequent encounter
T56.7X2A	Toxic effect of beryllium and its compounds, intentional self-harm, initial encounter
T56.7X2D	Toxic effect of beryllium and its compounds, intentional self-harm, subsequent encounter
T56.812A	Toxic effect of thallium, intentional self-harm, initial encounter
T56.812D	Toxic effect of thallium, intentional self-harm, subsequent encounter
T56.892A	Toxic effect of other metals, intentional self-harm, initial encounter
T56.892D	Toxic effect of other metals, intentional self-harm, subsequent encounter
T56.92XA	Toxic effect of unspecified metal, intentional self-harm, initial encounter
T56.92XD	Toxic effect of unspecified metal, intentional self-harm, subsequent encounter
T57.0X2A	Toxic effect of arsenic and its compounds, intentional self-harm, initial encounter
T57.0X2D	Toxic effect of arsenic and its compounds, intentional self-harm, subsequent encounter
T57.1X2A	Toxic effect of phosphorus and its compounds, intentional self-harm, initial encounter
T57.1X2D	Toxic effect of phosphorus and its compounds, intentional self-harm, subsequent encounter
T57.2X2A	Toxic effect of manganese and its compounds, intentional self-harm, initial encounter
T57.2X2D	Toxic effect of manganese and its compounds, intentional self-harm, subsequent encounter
T57.3X2A	Toxic effect of hydrogen cyanide, intentional self-harm, initial encounter
T57.3X2D	Toxic effect of hydrogen cyanide, intentional self-harm, subsequent encounter
T57.8X2A	Toxic effect of other specified inorganic substances, intentional self-harm, initial encounter

© 2024 Tenso K et al. *JAMA Network Open*.

T57.8X2D	Toxic effect of other specified inorganic substances, intentional self-harm, subsequent encounter
T57.92XA	Toxic effect of unspecified inorganic substance, intentional self-harm, initial encounter
T57.92XD	Toxic effect of unspecified inorganic substance, intentional self-harm, subsequent encounter
T58.02XA	Toxic effect of carbon monoxide from motor vehicle exhaust, intentional self-harm, initial encounter
T58.02XD	Toxic effect of carbon monoxide from motor vehicle exhaust, intentional self-harm, subsequent encounter
T58.12XA	Toxic effect of carbon monoxide from utility gas, intentional self-harm, initial encounter
T58.12XD	Toxic effect of carbon monoxide from utility gas, intentional self-harm, subsequent encounter
T58.2X2A	Toxic effect of carbon monoxide from incomplete combustion of other domestic fuels, intentional self-harm, initial encounter
T58.2X2D	Toxic effect of carbon monoxide from incomplete combustion of other domestic fuels, intentional self-harm, subsequent encounter
T58.8X2A	Toxic effect of carbon monoxide from other source, intentional self-harm, initial encounter
T58.8X2D	Toxic effect of carbon monoxide from other source, intentional self-harm, subsequent encounter
T58.92XA	Toxic effect of carbon monoxide from unspecified source, intentional self-harm, initial encounter
T58.92XD	Toxic effect of carbon monoxide from unspecified source, intentional self-harm, subsequent encounter
T59.0X2A	Toxic effect of nitrogen oxides, intentional self-harm, initial encounter
T59.0X2D	Toxic effect of nitrogen oxides, intentional self-harm, subsequent encounter
T59.1X2A	Toxic effect of sulfur dioxide, intentional self-harm, initial encounter
T59.1X2D	Toxic effect of sulfur dioxide, intentional self-harm, subsequent encounter
T59.2X2A	Toxic effect of formaldehyde, intentional self-harm, initial encounter
T59.2X2D	Toxic effect of formaldehyde, intentional self-harm, subsequent encounter
T59.3X2A	Toxic effect of lacrimogenic gas, intentional self-harm, initial encounter
T59.3X2D	Toxic effect of lacrimogenic gas, intentional self-harm, subsequent encounter
T59.4X2A	Toxic effect of chlorine gas, intentional self-harm, initial encounter
T59.4X2D	Toxic effect of chlorine gas, intentional self-harm, subsequent encounter
T59.5X2A	Toxic effect of fluorine gas and hydrogen fluoride, intentional self-harm, initial encounter
T59.5X2D	Toxic effect of fluorine gas and hydrogen fluoride, intentional self-harm, subsequent encounter
T59.6X2A	Toxic effect of hydrogen sulfide, intentional self-harm, initial encounter
T59.6X2D	Toxic effect of hydrogen sulfide, intentional self-harm, subsequent encounter
T59.7X2A	Toxic effect of carbon dioxide, intentional self-harm, initial encounter
T59.7X2D	Toxic effect of carbon dioxide, intentional self-harm, subsequent encounter
T59.812A	Toxic effect of smoke, intentional self-harm, initial encounter
T59.812D	Toxic effect of smoke, intentional self-harm, subsequent encounter
T59.892A	Toxic effect of other specified gases, fumes and vapors, intentional self-harm, initial encounter
T59.892D	Toxic effect of other specified gases, fumes and vapors, intentional self-harm, subsequent encounter
T59.92XA	Toxic effect of unspecified gases, fumes and vapors, intentional self-harm, initial encounter
T59.92XD	Toxic effect of unspecified gases, fumes and vapors, intentional self-harm, subsequent encounter
T60.0X2A	Toxic effect of organophosphate and carbamate insecticides, intentional self-harm, initial encounter
T60.0X2D	Toxic effect of organophosphate and carbamate insecticides, intentional self-harm, subsequent encounter

© 2024 Tenso K et al. *JAMA Network Open*.

T60.1X2A	Toxic effect of halogenated insecticides, intentional self-harm, initial encounter
T60.1X2D	Toxic effect of halogenated insecticides, intentional self-harm, subsequent encounter
T60.2X2A	Toxic effect of other insecticides, intentional self-harm, initial encounter
T60.2X2D	Toxic effect of other insecticides, intentional self-harm, subsequent encounter
T60.3X2A	Toxic effect of herbicides and fungicides, intentional self-harm, initial encounter
T60.3X2D	Toxic effect of herbicides and fungicides, intentional self-harm, subsequent encounter
T60.4X2A	Toxic effect of rodenticides, intentional self-harm, initial encounter
T60.4X2D	Toxic effect of rodenticides, intentional self-harm, subsequent encounter
T60.8X2A	Toxic effect of other pesticides, intentional self-harm, initial encounter
T60.8X2D	Toxic effect of other pesticides, intentional self-harm, subsequent encounter
T60.92XA	Toxic effect of unspecified pesticide, intentional self-harm, initial encounter
T60.92XD	Toxic effect of unspecified pesticide, intentional self-harm, subsequent encounter
T61.02XA	Ciguatera fish poisoning, intentional self-harm, initial encounter
T61.02XD	Ciguatera fish poisoning, intentional self-harm, subsequent encounter
T61.12XA	Scombroid fish poisoning, intentional self-harm, initial encounter
T61.12XD	Scombroid fish poisoning, intentional self-harm, subsequent encounter
T61.772A	Other fish poisoning, intentional self-harm, initial encounter
T61.772D	Other fish poisoning, intentional self-harm, subsequent encounter
T61.782A	Other shellfish poisoning, intentional self-harm, initial encounter
T61.782D	Other shellfish poisoning, intentional self-harm, subsequent encounter
T61.8X2A	Toxic effect of other seafood, intentional self-harm, initial encounter
T61.8X2D	Toxic effect of other seafood, intentional self-harm, subsequent encounter
T61.92XA	Toxic effect of unspecified seafood, intentional self-harm, initial encounter
T61.92XD	Toxic effect of unspecified seafood, intentional self-harm, subsequent encounter
T62.0X2A	Toxic effect of ingested mushrooms, intentional self-harm, initial encounter
T62.0X2D	Toxic effect of ingested mushrooms, intentional self-harm, subsequent encounter
T62.1X2A	Toxic effect of ingested berries, intentional self-harm, initial encounter
T62.1X2D	Toxic effect of ingested berries, intentional self-harm, subsequent encounter
T62.2X2A	Toxic effect of other ingested (parts of) plant(s), intentional self-harm, initial encounter
T62.2X2D	Toxic effect of other ingested (parts of) plant(s), intentional self-harm, subsequent encounter
T62.8X2A	Toxic effect of other specified noxious substances eaten as food, intentional self-harm, initial encounter
T62.8X2D	Toxic effect of other specified noxious substances eaten as food, intentional self-harm, subsequent encounter
T62.92XA	Toxic effect of unspecified noxious substance eaten as food, intentional self-harm, initial encounter
T62.92XD	Toxic effect of unspecified noxious substance eaten as food, intentional self-harm, subsequent encounter
T63.002A	Toxic effect of unspecified snake venom, intentional self-harm, initial encounter
T63.002D	Toxic effect of unspecified snake venom, intentional self-harm, subsequent encounter
T63.012A	Toxic effect of rattlesnake venom, intentional self-harm, initial encounter
T63.012D	Toxic effect of rattlesnake venom, intentional self-harm, subsequent encounter
T63.022A	Toxic effect of coral snake venom, intentional self-harm, initial encounter

© 2024 Tenso K et al. *JAMA Network Open*.

T63.022D	Toxic effect of coral snake venom, intentional self-harm, subsequent encounter
T63.032A	Toxic effect of taipan venom, intentional self-harm, initial encounter
T63.032D	Toxic effect of taipan venom, intentional self-harm, subsequent encounter
T63.042A	Toxic effect of cobra venom, intentional self-harm, initial encounter
T63.042D	Toxic effect of cobra venom, intentional self-harm, subsequent encounter
T63.062A	Toxic effect of venom of other North and South American snake, intentional self-harm, initial encounter
T63.062D	Toxic effect of venom of other North and South American snake, intentional self-harm, subsequent encounter
T63.072A	Toxic effect of venom of other Australian snake, intentional self-harm, initial encounter
T63.072D	Toxic effect of venom of other Australian snake, intentional self-harm, subsequent encounter
T63.082A	Toxic effect of venom of other African and Asian snake, intentional self-harm, initial encounter
T63.082D	Toxic effect of venom of other African and Asian snake, intentional self-harm, subsequent encounter
T63.092A	Toxic effect of venom of other snake, intentional self-harm, initial encounter
T63.092D	Toxic effect of venom of other snake, intentional self-harm, subsequent encounter
T63.112A	Toxic effect of venom of gila monster, intentional self-harm, initial encounter
T63.112D	Toxic effect of venom of gila monster, intentional self-harm, subsequent encounter
T63.122A	Toxic effect of venom of other venomous lizard, intentional self-harm, initial encounter
T63.122D	Toxic effect of venom of other venomous lizard, intentional self-harm, subsequent encounter
T63.192A	Toxic effect of venom of other reptiles, intentional self-harm, initial encounter
T63.192D	Toxic effect of venom of other reptiles, intentional self-harm, subsequent encounter
T63.2X2A	Toxic effect of venom of scorpion, intentional self-harm, initial encounter
T63.2X2D	Toxic effect of venom of scorpion, intentional self-harm, subsequent encounter
T63.302A	Toxic effect of unspecified spider venom, intentional self-harm, initial encounter
T63.302D	Toxic effect of unspecified spider venom, intentional self-harm, subsequent encounter
T63.312A	Toxic effect of venom of black widow spider, intentional self-harm, initial encounter
T63.312D	Toxic effect of venom of black widow spider, intentional self-harm, subsequent encounter
T63.322A	Toxic effect of venom of tarantula, intentional self-harm, initial encounter
T63.322D	Toxic effect of venom of tarantula, intentional self-harm, subsequent encounter
T63.332A	Toxic effect of venom of brown recluse spider, intentional self-harm, initial encounter
T63.332D	Toxic effect of venom of brown recluse spider, intentional self-harm, subsequent encounter
T63.392A	Toxic effect of venom of other spider, intentional self-harm, initial encounter
T63.392D	Toxic effect of venom of other spider, intentional self-harm, subsequent encounter
T63.412A	Toxic effect of venom of centipedes and venomous millipedes, intentional self-harm, initial encounter
T63.412D	Toxic effect of venom of centipedes and venomous millipedes, intentional self-harm, subsequent encounter
T63.422A	Toxic effect of venom of ants, intentional self-harm, initial encounter
T63.422D	Toxic effect of venom of ants, intentional self-harm, subsequent encounter
T63.432A	Toxic effect of venom of caterpillars, intentional self-harm, initial encounter
T63.432D	Toxic effect of venom of caterpillars, intentional self-harm, subsequent encounter
T63.442A	Toxic effect of venom of bees, intentional self-harm, initial encounter
T63.442D	Toxic effect of venom of bees, intentional self-harm, subsequent encounter

© 2024 Tenso K et al. *JAMA Network Open*.

T63.452A	Toxic effect of venom of hornets, intentional self-harm, initial encounter
T63.452D	Toxic effect of venom of hornets, intentional self-harm, subsequent encounter
T63.462A	Toxic effect of venom of wasps, intentional self-harm, initial encounter
T63.462D	Toxic effect of venom of wasps, intentional self-harm, subsequent encounter
T63.482A	Toxic effect of venom of other arthropod, intentional self-harm, initial encounter
T63.482D	Toxic effect of venom of other arthropod, intentional self-harm, subsequent encounter
T63.512A	Toxic effect of contact with stingray, intentional self-harm, initial encounter
T63.512D	Toxic effect of contact with stingray, intentional self-harm, subsequent encounter
T63.592A	Toxic effect of contact with other venomous fish, intentional self-harm, initial encounter
T63.592D	Toxic effect of contact with other venomous fish, intentional self-harm, subsequent encounter
T63.612A	Toxic effect of contact with Portugese Man-o-war, intentional self-harm, initial encounter
T63.612D	Toxic effect of contact with Portugese Man-o-war, intentional self-harm, subsequent encounter
T63.622A	Toxic effect of contact with other jellyfish, intentional self-harm, initial encounter
T63.622D	Toxic effect of contact with other jellyfish, intentional self-harm, subsequent encounter
T63.632A	Toxic effect of contact with sea anemone, intentional self-harm, initial encounter
T63.632D	Toxic effect of contact with sea anemone, intentional self-harm, subsequent encounter
T63.692A	Toxic effect of contact with other venomous marine animals, intentional self-harm, initial encounter
T63.692D	Toxic effect of contact with other venomous marine animals, intentional self-harm, subsequent encounter
T63.712A	Toxic effect of contact with venomous marine plant, intentional self-harm, initial encounter
T63.712D	Toxic effect of contact with venomous marine plant, intentional self-harm, subsequent encounter
T63.792A	Toxic effect of contact with other venomous plant, intentional self-harm, initial encounter
T63.792D	Toxic effect of contact with other venomous plant, intentional self-harm, subsequent encounter
T63.812A	Toxic effect of contact with venomous frog, intentional self-harm, initial encounter
T63.812D	Toxic effect of contact with venomous frog, intentional self-harm, subsequent encounter
T63.822A	Toxic effect of contact with venomous toad, intentional self-harm, initial encounter
T63.822D	Toxic effect of contact with venomous toad, intentional self-harm, subsequent encounter
T63.832A	Toxic effect of contact with other venomous amphibian, intentional self-harm, initial encounter
T63.832D	Toxic effect of contact with other venomous amphibian, intentional self-harm, subsequent encounter
T63.892A	Toxic effect of contact with other venomous animals, intentional self-harm, initial encounter
T63.892D	Toxic effect of contact with other venomous animals, intentional self-harm, subsequent encounter
T63.92XA	Toxic effect of contact with unspecified venomous animal, intentional self-harm, initial encounter
T63.92XD	Toxic effect of contact with unspecified venomous animal, intentional self-harm, subsequent encounter
T64.02XA	Toxic effect of aflatoxin, intentional self-harm, initial encounter
T64.02XD	Toxic effect of aflatoxin, intentional self-harm, subsequent encounter
T64.82XA	Toxic effect of other mycotoxin food contaminants, intentional self-harm, initial encounter
T64.82XD	Toxic effect of other mycotoxin food contaminants, intentional self-harm, subsequent encounter
T65.0X2A	Toxic effect of cyanides, intentional self-harm, initial encounter
T65.0X2D	Toxic effect of cyanides, intentional self-harm, subsequent encounter
T65.1X2A	Toxic effect of strychnine and its salts, intentional self-harm, initial encounter

© 2024 Tenso K et al. *JAMA Network Open*.

T65.1X2D	Toxic effect of strychnine and its salts, intentional self-harm, subsequent encounter
T65.212A	Toxic effect of chewing tobacco, intentional self-harm, initial encounter
T65.212D	Toxic effect of chewing tobacco, intentional self-harm, subsequent encounter
T65.222A	Toxic effect of tobacco cigarettes, intentional self-harm, initial encounter
T65.222D	Toxic effect of tobacco cigarettes, intentional self-harm, subsequent encounter
T65.292A	Toxic effect of other tobacco and nicotine, intentional self-harm, initial encounter
T65.292D	Toxic effect of other tobacco and nicotine, intentional self-harm, subsequent encounter
T65.3X2A	Toxic effect of nitroderivatives and aminoderivatives of benzene and its homologues, intentional self-harm, initial encounter
T65.3X2D	Toxic effect of nitroderivatives and aminoderivatives of benzene and its homologues, intentional self-harm, subsequent encounter
T65.4X2A	Toxic effect of carbon disulfide, intentional self-harm, initial encounter
T65.4X2D	Toxic effect of carbon disulfide, intentional self-harm, subsequent encounter
T65.5X2A	Toxic effect of nitroglycerin and other nitric acids and esters, intentional self-harm, initial encounter
T65.5X2D	Toxic effect of nitroglycerin and other nitric acids and esters, intentional self-harm, subsequent encounter
T65.6X2A	Toxic effect of paints and dyes, not elsewhere classified, intentional self-harm, initial encounter
T65.6X2D	Toxic effect of paints and dyes, not elsewhere classified, intentional self-harm, subsequent encounter
T65.812A	Toxic effect of latex, intentional self-harm, initial encounter
T65.812D	Toxic effect of latex, intentional self-harm, subsequent encounter
T65.822A	Toxic effect of harmful algae and algae toxins, intentional self-harm, initial encounter
T65.822D	Toxic effect of harmful algae and algae toxins, intentional self-harm, subsequent encounter
T65.832A	Toxic effect of fiberglass, intentional self-harm, initial encounter
T65.832D	Toxic effect of fiberglass, intentional self-harm, subsequent encounter
T65.892A	Toxic effect of other specified substances, intentional self-harm, initial encounter
T65.892D	Toxic effect of other specified substances, intentional self-harm, subsequent encounter
T65.92XA	Toxic effect of unspecified substance, intentional self-harm, initial encounter
T65.92XD	Toxic effect of unspecified substance, intentional self-harm, subsequent encounter
T71.112A	Asphyxiation due to smothering under pillow, intentional self-harm, initial encounter
T71.112D	Asphyxiation due to smothering under pillow, intentional self-harm, subsequent encounter
T71.122A	Asphyxiation due to plastic bag, intentional self-harm, initial encounter
T71.122D	Asphyxiation due to plastic bag, intentional self-harm, subsequent encounter
T71.132A	Asphyxiation due to being trapped in bed linens, intentional self-harm, initial encounter
T71.132D	Asphyxiation due to being trapped in bed linens, intentional self-harm, subsequent encounter
T71.152A	Asphyxiation due to smothering in furniture, intentional self-harm, initial encounter
T71.152D	Asphyxiation due to smothering in furniture, intentional self-harm, subsequent encounter
T71.162A	Asphyxiation due to hanging, intentional self-harm, initial encounter
T71.162D	Asphyxiation due to hanging, intentional self-harm, subsequent encounter
T71.192A	Asphyxiation due to mechanical threat to breathing due to other causes, intentional self-harm, initial encounter
T71.192D	Asphyxiation due to mechanical threat to breathing due to other causes, intentional self-harm, subsequent encounter
T71.222A	Asphyxiation due to being trapped in a car trunk, intentional self-harm, initial encounter

© 2024 Tenso K et al. *JAMA Network Open*.

T71.222D	Asphyxiation due to being trapped in a car trunk, intentional self-harm, subsequent encounter
T71.232A	Asphyxiation due to being trapped in a (discarded) refrigerator, intentional self-harm, initial encounter
T71.232D	Asphyxiation due to being trapped in a (discarded) refrigerator, intentional self-harm, subsequent encounter
X71.0XXA	Intentional self-harm by drowning and submersion while in bathtub, initial encounter
X71.0XXD	Intentional self-harm by drowning and submersion while in bathtub, subsequent encounter
X71.1XXA	Intentional self-harm by drowning and submersion while in swimming pool, initial encounter
X71.1XXD	Intentional self-harm by drowning and submersion while in swimming pool, subsequent encounter
X71.2XXA	Intentional self-harm by drowning and submersion after jump into swimming pool, initial encounter
X71.2XXD	Intentional self-harm by drowning and submersion after jump into swimming pool, subsequent encounter
X71.3XXA	Intentional self-harm by drowning and submersion in natural water, initial encounter
X71.3XXD	Intentional self-harm by drowning and submersion in natural water, subsequent encounter
X71.8XXA	Other intentional self-harm by drowning and submersion, initial encounter
X71.8XXD	Other intentional self-harm by drowning and submersion, subsequent encounter
X71.9XXA	Intentional self-harm by drowning and submersion, unspecified, initial encounter
X71.9XXD	Intentional self-harm by drowning and submersion, unspecified, subsequent encounter
X72.XXXA	Intentional self-harm by handgun discharge, initial encounter
X72.XXXD	Intentional self-harm by handgun discharge, subsequent encounter
X73.0XXA	Intentional self-harm by shotgun discharge, initial encounter
X73.0XXD	Intentional self-harm by shotgun discharge, subsequent encounter
X73.1XXA	Intentional self-harm by hunting rifle discharge, initial encounter
X73.1XXD	Intentional self-harm by hunting rifle discharge, subsequent encounter
X73.2XXA	Intentional self-harm by machine gun discharge, initial encounter
X73.2XXD	Intentional self-harm by machine gun discharge, subsequent encounter
X73.8XXA	Intentional self-harm by other larger firearm discharge, initial encounter
X73.8XXD	Intentional self-harm by other larger firearm discharge, subsequent encounter
X73.9XXA	Intentional self-harm by unspecified larger firearm discharge, initial encounter
X73.9XXD	Intentional self-harm by unspecified larger firearm discharge, subsequent encounter
X74.01XA	Intentional self-harm by airgun, initial encounter
X74.01XD	Intentional self-harm by airgun, subsequent encounter
X74.02XA	Intentional self-harm by paintball gun, initial encounter
X74.02XD	Intentional self-harm by paintball gun, subsequent encounter
X74.09XA	Intentional self-harm by other gas, air or spring-operated gun, initial encounter
X74.09XD	Intentional self-harm by other gas, air or spring-operated gun, subsequent encounter
X74.8XXA	Intentional self-harm by other firearm discharge, initial encounter
X74.8XXD	Intentional self-harm by other firearm discharge, subsequent encounter
X74.9XXA	Intentional self-harm by unspecified firearm discharge, initial encounter
X74.9XXD	Intentional self-harm by unspecified firearm discharge, subsequent encounter
X75.XXXA	Intentional self-harm by explosive material, initial encounter
X75.XXXD	Intentional self-harm by explosive material, subsequent encounter

© 2024 Tenso K et al. *JAMA Network Open*.

X76.XXXA	Intentional self-harm by smoke, fire and flames, initial encounter
X76.XXXD	Intentional self-harm by smoke, fire and flames, subsequent encounter
X77.0XXA	Intentional self-harm by steam or hot vapors, initial encounter
X77.0XXD	Intentional self-harm by steam or hot vapors, subsequent encounter
X77.1XXA	Intentional self-harm by hot tap water, initial encounter
X77.1XXD	Intentional self-harm by hot tap water, subsequent encounter
X77.2XXA	Intentional self-harm by other hot fluids, initial encounter
X77.2XXD	Intentional self-harm by other hot fluids, subsequent encounter
X77.3XXA	Intentional self-harm by hot household appliances, initial encounter
X77.3XXD	Intentional self-harm by hot household appliances, subsequent encounter
X77.8XXA	Intentional self-harm by other hot objects, initial encounter
X77.8XXD	Intentional self-harm by other hot objects, subsequent encounter
X77.9XXA	Intentional self-harm by unspecified hot objects, initial encounter
X77.9XXD	Intentional self-harm by unspecified hot objects, subsequent encounter
X78.0XXA	Intentional self-harm by sharp glass, initial encounter
X78.0XXD	Intentional self-harm by sharp glass, subsequent encounter
X78.1XXA	Intentional self-harm by knife, initial encounter
X78.1XXD	Intentional self-harm by knife, subsequent encounter
X78.2XXA	Intentional self-harm by sword or dagger, initial encounter
X78.2XXD	Intentional self-harm by sword or dagger, subsequent encounter
X78.8XXA	Intentional self-harm by other sharp object, initial encounter
X78.8XXD	Intentional self-harm by other sharp object, subsequent encounter
X78.9XXA	Intentional self-harm by unspecified sharp object, initial encounter
X78.9XXD	Intentional self-harm by unspecified sharp object, subsequent encounter
X79.XXXA	Intentional self-harm by blunt object, initial encounter
X79.XXXD	Intentional self-harm by blunt object, subsequent encounter
X80.XXXA	Intentional self-harm by jumping from a high place, initial encounter
X80.XXXD	Intentional self-harm by jumping from a high place, subsequent encounter
X81.0XXA	Intentional self-harm by jumping or lying in front of motor vehicle, initial encounter
X81.0XXD	Intentional self-harm by jumping or lying in front of motor vehicle, subsequent encounter
X81.1XXA	Intentional self-harm by jumping or lying in front of (subway) train, initial encounter
X81.1XXD	Intentional self-harm by jumping or lying in front of (subway) train, subsequent encounter
X81.8XXA	Intentional self-harm by jumping or lying in front of other moving object, initial encounter
X81.8XXD	Intentional self-harm by jumping or lying in front of other moving object, subsequent encounter
X82.8XXA	Other intentional self-harm by crashing of motor vehicle, initial encounter
X82.8XXD	Other intentional self-harm by crashing of motor vehicle, subsequent encounter
X83.0XXA	Intentional self-harm by crashing of aircraft, initial encounter
X83.0XXD	Intentional self-harm by crashing of aircraft, subsequent encounter
X83.1XXA	Intentional self-harm by electrocution, initial encounter
X83.1XXD	Intentional self-harm by electrocution, subsequent encounter
X83.2XXA	Intentional self-harm by exposure to extremes of cold, initial encounter

© 2024 Tenso K et al. *JAMA Network Open*.

X83.2XXD	Intentional self-harm by exposure to extremes of cold, subsequent encounter
X83.8XXA	Intentional self-harm by other specified means, initial encounter
X83.8XXD	Intentional self-harm by other specified means, subsequent encounter

© 2024 Tenso K et al. *JAMA Network Open*.

eAppendix 4. Falsification test

A key concern was that broadband speed and availability might directly affect mental health and SREs by increasing feelings of loneliness and social isolation. To investigate this, we divided the data into quartiles based on virtual care utilization, comparing the lowest quartile (those with no virtual mental healthcare use) to the highest quartile (those with extensive use of virtual mental healthcare). Both groups showed variation in broadband speed and provider availability. We then carried out a stratified regression analysis with SREs as the outcome, accounting for all previously included independent variables and integrating broadband metrics as covariates.

Our hypothesis was that, if broadband accessibility correlates with social isolation, and is a confounding effect on SREs, we would expect a statistically significant positive association between broadband speed and suicide attempts in a group devoid of virtual care utilization. However, if this correlation weakens in the high virtual care group, it would suggest that virtual care mitigates the effects of isolation.

The results of this falsification test indicated no statistically significant relationship between broadband variability and SREs, which supports the exclusion restriction and confirms the validity of our instrument.

© 2024 Tenso K et al. *JAMA Network Open*.

eAppendix 5. Heckman correction

The reason for considering Heckman modeling is that suicide-related events are only recorded for individuals who engage with the healthcare system, leading to partial observability of these events. Additionally, pre-existing mental health needs are likely to affect the use of mental health services, and suicide-related events can only be observed in patients receiving care in VHA facilities, introducing a selection bias concern. Therefore, we integrated the Heckman model with the IVprobit approach to address endogeneity issues stemming from partial observability and to correct for reverse causality and potential confounding from unobserved variables.

Initially, we estimated the first stage of the Heckman model on the full sample (including observations with no mental health utilization), followed by the estimation of the IVprobit model in the second stage that only included observations with mental health use. To compute the Inverse Mills Ratio (IMR), we estimated a Probit model with the dependent variable being individual-level VHA outpatient mental health utilization. Subsequently, we incorporated the IMR into the quasi-experimental instrumental variables IVprobit models. Individual-level distance from the VHA facility was included as an exogenous predictor in the first stage of the Heckman model but excluded from the IV probit models. While distance from any VHA facility is likely to be linked with the availability and utilization of mental health care, it is not anticipated to directly affect suicide-related outcomes or unobserved factors.

The examination for Heckman correction demonstrated that partial observability does not present a concern for our analysis. Specifically, the p-value of Heckman residuals (IMR) was not statistically significant in the second stage ($p = 0.342$) of IVprobit. This indicates that unobservable factors influencing the receipt of mental health care at the VHA are not correlated with non-fatal suicide attempts. Consequently, there is no requirement for Heckman correction. The analysis continued with IVprobit, utilizing both distance and broadband speed as instruments.

© 2024 Tenso K et al. *JAMA Network Open*.

eAppendix 6. Average virtual care use by demographic subgroups

Characteristic	Mean virtual care use percentage
Sex	
Male	48.5
Female	42.7
Ethnicity	
Hispanic	46.9
Non-Hispanic	42.8
Race	
American Indian or Alaska Native	40.7
Asian	48.1
Black	44.3
Native Hawaiian or Other Pacific Islander	46.2
More Than One Race	46.0
Race Unknown	44.2

© 2024 Tenso K et al. *JAMA Network Open*.

eAppendix 7. Probit and IVProbit second stage marginal effects

Variable	Probit	IVProbit 2nd stage
% of virtual mental health visits	-0.0001	-0.0007
Age in years	-0.0009	-0.0006
Sex (ref. male)		
Female	-0.0012	0.0026
Race (ref. white)		
American Indian or Alaska native	0.0099	0.0062
Asian	-0.0012	-0.0016
Black	-0.0017	-0.0034
Native Hawaiian or Other Pacific Islander	-0.0077	-0.0089
More than one race	-0.0048	-0.0050
Race unknown	-0.0001	-0.0010
Ethnicity (ref. non-Hispanic)		
Hispanic	-0.0005	-0.0002
Ethnicity unknown	-0.0010	-0.0019
Marital status (ref. married)		
Single/Never married	0.0023	0.0012
Divorced/Separated/Widowed	0.0026	0.0008
Marital status unknown	0.0020	0.0034
Priority status (ref. groups 1–3: service-connected disability, no co-pays)		
4–6 (no service-connected disability, no copays)	0.0090	0.0035
7–8 (no service-connected disability, have copays)	0.0145	0.0119
Priority status unknown	0.0137	-0.0042
Rurality (ref. not rural or islands)		
Rural	0.0015	0.0020
Health insurance coverage as proportion of facility enrollees (ref. no insurance)		
Comprehensive coverage	-0.0404	-0.1092
Some coverage	-0.0820	-0.1149
Insurance unknown	0.1016	0.3642
Household income as proportion of facility enrollees (ref. >= USD 75,000)		
Below USD 20,000	0.0029	-0.1007

© 2024 Tenso K et al. *JAMA Network Open*.

USD 20,000–50,000 (excluded)	-0.0987	-0.1529
USD 50,000–75,000 (excluded)	-0.0861	-0.1079
Income unknown	-0.0232	-0.1518
Months since separation	-0.0002	0.0001
COVID-19 mortality per 10,000	0.0000	0.0005

© 2024 Tenso K et al. *JAMA Network Open*.

eAppendix 8. Sensitivity analysis: one month lead in suicide-related events

Variable	(1) IV probit 1st stage[a]	(2) IV probit 2nd stage[a]
% of virtual mental health visits		-0.0179
		(0.0044)
		0.0001
% of county with >= 3 Internet providers w. >= 100/Mbps speed	0.0927	
	(0.0184)	
	0.0000	
Age in years	0.4500	-0.0111
	(0.0412)	(0.0079)
	0.0000	0.1610
Sex (ref. male)		
Female	7.1172	0.0837
	(0.7218)	(0.0587)
	0.0000	0.1536
Race (ref. white)		
American Indian or Alaska native	-4.3751	0.0767
	(3.1663)	(0.1220)
	0.1670	0.5297
Asian	-0.5746	-0.0399
	(1.9620)	(0.0900)
	0.7696	0.6573
Black	-3.7536	-0.1045
	(0.8286)	(0.0446)
	0.0000	0.0192
Native Hawaiian or Other Pacific Islander	-2.7532	-0.2243
	(2.7316)	(0.1732)
	0.3135	0.1953
More than one race	-1.1809	-0.1427
	(2.0714)	(0.1159)
	0.5686	0.2180
Race unknown	-2.0883	-0.0337
	(1.0686)	(0.0535)
	0.0507	0.5295
Ethnicity (ref. non-Hispanic)		
Hispanic	0.7264	-0.0085
	(1.0209)	(0.0500)
	0.4768	0.8646
Ethnicity unknown	-1.1620	-0.0461

© 2024 Tenso K et al. *JAMA Network Open*.

Ethnicity unknown	(1.0163)	(0.0501)
	0.2529	0.3577
Marital status (ref. married)		
Single/Never married	-1.9753	0.0339
	(0.8434)	(0.0448)
	0.0192	0.4491
Divorced/Separated/Widowed	-3.0180	0.0038
	(0.9642)	(0.0557)
	0.0017	0.9459
Marital status unknown	2.1820	0.0854
	(1.6635)	(0.0762)
	0.1896	0.2622
Priority status (ref. groups 1–3: service-connected disability, no co-pays)		
4–6 (no service-connected disability, no copays)	-6.8392	0.0087
	(1.3411)	(0.0877)
	0.0000	0.9210
7–8 (no service-connected disability, have copays)	-2.2999	0.2162
	(3.1324)	(0.1303)
	0.4628	0.0970
Priority status unknown	-24.8165	-0.1780
	(6.4772)	(0.2723)
	0.0001	0.5133
Rurality (ref. not rural or islands)		
Rural	-0.2559	0.0492
	(1.0392)	(0.0475)
	0.8055	0.3005
Drive distance to closest VHA primary care facility	0.0593	0.0006
	(0.0387)	(0.0015)
	0.1255	0.7080
Health insurance coverage as proportion of facility enrollees (ref. no insurance)		
Comprehensive coverage	-106.3066	-3.0552
	(38.4532)	(1.7770)
	0.0057	0.0856
Some coverage	-92.5067	-4.1264
	(45.1991)	(2.1011)
	0.0407	0.0495
Insurance unknown	432.7729	1.9784
	(239.2273)	(13.0091)

© 2024 Tenso K et al. *JAMA Network Open*.

Insurance unknown	0.0704	0.8791
Household income as proportion of facility enrollees (ref. >= USD 75,000)		
Below USD 20,000	-149.9685	-3.9881
	(43.8858)	(2.2642)
	0.0006	0.0782
USD 20,000–50,000 (excluded)	-54.4290	-4.3348
	(39.2802)	(1.9024)
	0.1659	0.0227
USD 50,000–75,000 (excluded)	-21.1584	-3.3802
	(36.0148)	(1.8687)
	0.5569	0.0705
Income unknown	-206.3060	-2.6408
	(69.7159)	(3.2807)
	0.0031	0.4209
Months since separation	1.1284	0.0151
	(0.0861)	(0.0081)
	0.0000	0.0622
COVID-19 mortality per 10,000	1.0268	0.0159
	(0.0589)	(0.0066)
	0.0000	0.0166
Observations	49,122	49,122
Station FE	YES	YES
Month FE	YES	YES

[a] robust standard errors are between parentheses, followed by p-values.

© 2024 Tenso K et al. *JAMA Network Open*.

eAppendix 9. Table 4. Sensitivity analysis: exclusion of March, April, and May 2020 data

Variable	(1)<br>IV probit 1st stage[a]	(2)<br>IV probit 2nd stage[a]
% of virtual mental health visits		-0.0178
		(0.0054)
		0.0010
% of county with >= 3 Internet providers w. >= 100/Mbps speed	0.0708	
	(0.0172)	
	0.0000	
Age in years	0.4406	-0.0122
	(0.0405)	(0.0093)
	0.0000	0.1885
Sex (ref. male)		
Female	7.0040	0.0807
	(0.7099)	(0.0645)
	0.0000	0.2109
Race (ref. white)		
Black	-2.8856	-0.0712
	(0.8164)	(0.0423)
	0.0004	0.0925
Asian	-0.1251	-0.0205
	(1.9328)	(0.0889)
	0.9484	0.8175
More than one race	-0.7341	-0.2344
	(2.0272)	(0.1313)
	0.7172	0.0743
American Indian or Alaska native	-3.9892	0.0347
	(3.1458)	(0.1245)
	0.2048	0.7803
Native Hawaiian or Other Pacific Islander	-3.1837	-0.2150
	(2.6946)	(0.1789)
	0.2374	0.2293
Race unknown	-1.4634	-0.0117
	(1.0294)	(0.0530)
	0.1551	0.8252
Ethnicity (ref. non-Hispanic)		
Hispanic	0.5178	0.0118
	(1.0014)	(0.0484)
	0.6051	0.8079
Ethnicity unknown	-2.0605	-0.0622

© 2024 Tenso K et al. *JAMA Network Open*.

Ethnicity unknown	(0.9447)	(0.0452)
	0.0292	0.1687
Marital status (ref. married)		
Single/Never married	-2.2377	0.0264
	(0.8342)	(0.0487)
	0.0073	0.5877
Divorced/Separated/Widowed	-3.2545	0.0271
	(0.9390)	(0.0652)
	0.0005	0.6772
Marital status unknown	1.8274	0.0866
	(1.5578)	(0.0727)
	0.2408	0.2337
Priority status (ref. groups 1–3: service-connected disability, no co-pays)		
4–6 (no service-connected disability, no copays)	-7.3986	-0.0035
	(1.2864)	(0.0980)
	0.0000	0.9719
7–8 (no service-connected disability, have copays)	-3.3285	0.2167
	(2.8029)	(0.1429)
	0.2350	0.1294
Priority status unknown	-28.2039	-0.2388
	(4.4589)	(0.2961)
	0.0000	0.4200
Rurality (ref. not rural or islands)	1.1133	0.0362
Rural	(1.0013)	(0.0440)
	0.2662	0.4103
Drive distance to closest VHA primary care facility	0.0733	0.0001
	(0.0355)	(0.0016)
	0.0389	0.9434
Health insurance coverage as proportion of facility enrollees (ref. no insurance)		
Comprehensive coverage	-128.8262	-1.6624
	(34.7942)	(1.9323)
	0.0002	0.3896
Some coverage	-92.2578	-1.3503
	(42.3842)	(2.0726)
	0.0295	0.5147
Insurance unknown	441.8803	11.8170
	(222.1595)	(10.6963)
	0.0467	0.2693

© 2024 Tenso K et al. *JAMA Network Open*.

Household income as proportion of facility enrollees (ref. >= USD 75,000)		
Below USD 20,000	-28.2546	0.1795
	(41.8681)	(2.2782)
	0.4998	0.9372
USD 20,000–50,000 (excluded)	12.6643	-1.4742
	(37.5364)	(1.8100)
	0.7358	0.4154
USD 50,000–75,000 (excluded)	9.3707	-1.0787
	(33.4773)	(1.7013)
	0.7795	0.5260
Income unknown	32.4725	0.2267
	(65.0317)	(2.9635)
	0.6175	0.9390
Months since separation	0.8772	0.0114
	(0.0790)	(0.0077)
	0.0000	0.1371
COVID-19 mortality per 10,000	0.4482	0.0072
	(0.0586)	(0.0044)
	0.0000	0.1030
Observations	54,466	54,466
Station FE	YES	YES
Month FE	YES	YES

[a] robust standard errors are between parentheses, followed by p-values.

© 2024 Tenso K et al. *JAMA Network Open*.
