# Supplementary material for: Virtual Mental Health Care and Suicide-Related Events
Source: JAMA Netw Open. 2024 Nov 5;7(11):e2443054. doi: 10.1001/jamanetworkopen.2024.43054 (PMC11539012; doi:10.1001/jamanetworkopen.2024.43054)
Supplement: Supplement 2. — Data Sharing Statement [file jamanetwopen-e2443054-s002.pdf]

Data Sharing Statement

Tenso. Comparative Effectiveness of Virtual Mental Health Care on Suicide-Related Events. *JAMA Netw Open*. Published November 05, 2024. doi:10.1001/jamanetworkopen.2024.43054

Data
Data available: No
